# Supplementary material for: Genetic approaches to the conservation of migratory bats: a study of the eastern red bat (Lasiurus borealis)
Source: PeerJ. 2015 May 28;3:e983. doi: 10.7717/peerj.983 (PMC4451038; doi:10.7717/peerj.983)
Supplement: Table S1 — Samples included in the primary dataset used in most analyses (samples collected between mainly between 2002 and 2006, but including samples from 1986 to 2007; dataset = 1) are listed first, sorted by population (in the same order as Table 1), followed by samples sequenced at HV2 but not included in site-level analyses. Samples included only in msvar analyses (2002 samples only, dataset = 2; 2010 samples only, dataset = 3) are subsequently listed, sorted by state and county. Note that samples included in dataset 1 may have also been included in dataset 2 (2002 only) if they were collected in 2002. Genbank accession numbers are provided for all unique sequences for HV2. For CHY, as a diploid nuclear marker, phased allelic sequences for each individual were submitted to Genbank, and so there are two accession numbers per individual sequenced. Sex (m, male; f, female), reproductive condition (RC: p, pregnant; l, lactating; pl, postlactating; s, scrotal; nr, non-reproductive, ns, non-scrotal), and age (a, adult; j, juvenile) are provided when available. [file peerj-03-983-s001.docx]

Table S1. Collection information for samples included in our study. Samples included in the primary dataset used in most analyses (samples collected between mainly between 2002-2006, but including samples from 1986-2007; dataset = 1) are listed first, sorted by population (in the same order as Table 1), followed by samples sequenced at HV2 but not included in site-level analyses. Samples included only in msvar analyses (2002 samples only, dataset = 2; 2010 samples only, dataset = 3) are subsequently listed, sorted by state and county. Note that samples included in dataset 1 may have also been included in dataset 2 (2002 only) if they were collected in 2002. Genbank accession numbers are provided for all unique sequences for HV2. For CHY, as a diploid nuclear marker, phased allelic sequences for each individual were submitted to Genbank, and so there are two accession numbers per individual sequenced. Sex (m=male, f=female), reproductive condition (RC: p=pregnant, l=lactating, pl=postlactating, s=scrotal, nr=non-reproductive, ns=non-scrotal), and age (a=adult, j=juvenile) are provided when available.

| Sample ID | Data Set | Genbank HV2 | Genbank CHY | Collection Date | Province or State | County | Country | Sex | RC | Age | Collected By |
| --- | --- | --- | --- | --- | --- | --- | --- | --- | --- | --- | --- |
| RP-01 | 1,2 | KR337228 | KR362438, KR362439 | 16-May-02 | Arkansas | Saline | USA | m |  | a | Roger Perry |
| RP-07 | 1,2 |  | KR362440, KR362441 | 12-Jun-02 | Arkansas | Saline | USA | f | l | a | Roger Perry |
| RP-09 | 1,2 | KR337229 | KR362442, KR362443 | 12-Jun-02 | Arkansas | Saline | USA | f | l | a | Roger Perry |
| RP-11 | 1,2 | KR337230 | KR362444, KR362445 | 19-Jun-02 | Arkansas | Saline | USA | f | l | a | Roger Perry |
| RP-12 | 1,2 | KR337231 |  | 12-Jun-02 | Arkansas | Saline | USA | m |  | a | Roger Perry |
| RP-14 | 1,2 |  |  | 13-Jun-02 | Arkansas | Saline | USA | f | l | a | Roger Perry |
| RP-15 | 1,2 | KR337232 | KR362446, KR362447 | 19-Jun-02 | Arkansas | Saline | USA | f | l | a | Roger Perry |
| RP-18 | 1,2 |  | KR362448, KR362449 | 27-Jun-02 | Arkansas | Saline | USA | m |  | a | Roger Perry |
| RP-19 | 1,2 | KR337233 | KR362450, KR362451 | 08-Jul-02 | Arkansas | Saline | USA | f | l | a | Roger Perry |
| RP-20 | 1,2 |  | KR362452, KR362453 | 08-Jul-02 | Arkansas | Saline | USA | f | pl | a | Roger Perry |
| RP-21 | 1,2 | KR337234 |  | 08-Jul-02 | Arkansas | Saline | USA | m |  | j | Roger Perry |
| RP-23 | 1,2 |  |  | 08-Jul-02 | Arkansas | Saline | USA | m |  | j | Roger Perry |
| RP-24 | 1,2 |  |  | 08-Jul-02 | Arkansas | Saline | USA | f |  | j | Roger Perry |
| RP-25 | 1,2 | KR337235 |  | 08-Jul-02 | Arkansas | Saline | USA | m |  | a | Roger Perry |
| RP-26 | 1,2 |  |  | 08-Jul-02 | Arkansas | Saline | USA | m |  | j | Roger Perry |
| RP-27 | 1,2 | KR337236 |  | 08-Jul-02 | Arkansas | Saline | USA | f |  | j | Roger Perry |
| RP-28 | 1,2 |  |  | 09-Jul-02 | Arkansas | Saline | USA | f | l | a | Roger Perry |
| RP-29 | 1,2 | KR337237 |  | 09-Jul-02 | Arkansas | Saline | USA | f | l | a | Roger Perry |
| RP-30 | 1,2 |  |  | 09-Jul-02 | Arkansas | Saline | USA | m |  | j | Roger Perry |
| RP-31 | 1,2 |  |  | 09-Jul-02 | Arkansas | Saline | USA | m |  | j | Roger Perry |
| RP-32 | 1,2 | KR337238 |  | 09-Jul-02 | Arkansas | Saline | USA | m |  | j | Roger Perry |
| RP-33 | 1,2 | KR337239 |  | 09-Jul-02 | Arkansas | Saline | USA | f | nr | a | Roger Perry |
| RP-35 | 1,2 |  |  | 10-Jul-02 | Arkansas | Saline | USA | f |  | j | Roger Perry |
| RP-36 | 1,2 | KR337240 |  | 10-Jul-02 | Arkansas | Saline | USA | m |  | j | Roger Perry |
| RP-37 | 1,2 |  |  | 10-Jul-02 | Arkansas | Saline | USA | m |  | j | Roger Perry |
| RP-39 | 1,2 |  |  | 10-Jul-02 | Arkansas | Saline | USA | m |  | j | Roger Perry |
| RP-44 | 1,2 |  |  | 22-Jul-02 | Arkansas | Saline | USA | m |  | j | Roger Perry |
| RP-45 | 1,2 |  |  | 22-Jul-02 | Arkansas | Saline | USA | m |  | j | Roger Perry |
| RP-47 | 1,2 |  |  | 22-Jul-02 | Arkansas | Saline | USA | m |  | j | Roger Perry |
| RP-48 | 1,2 |  |  | 22-Jul-02 | Arkansas | Saline | USA | m |  | j | Roger Perry |
| RP-49 | 1,2 |  |  | 22-Jul-02 | Arkansas | Saline | USA | m |  | j | Roger Perry |
| RP-50 | 1,2 |  |  | 23-Jul-02 | Arkansas | Saline | USA | m |  | a | Roger Perry |
| RP-53 | 1,2 |  |  | 29-Jul-02 | Arkansas | Saline | USA | f |  | j | Roger Perry |
| RP-54 | 1,2 |  |  | 29-Jul-02 | Arkansas | Saline | USA | m |  | j | Roger Perry |
| RP-55 | 1,2 |  |  | 29-Jul-02 | Arkansas | Saline | USA | f |  | a | Roger Perry |
| RP-56 | 1,2 |  |  | 29-Jul-02 | Arkansas | Saline | USA | f |  | j | Roger Perry |
| RP-58 | 1,2 |  |  | 29-Jul-02 | Arkansas | Saline | USA | f |  | j | Roger Perry |
| RP-60 | 1,2 |  |  | 29-Jul-02 | Arkansas | Saline | USA | m |  | j | Roger Perry |
| RP-61 | 1,2 | KR337241 |  | 29-Jul-02 | Arkansas | Saline | USA | m |  | a | Roger Perry |
| AM-09 | 1,2 | KR337115 |  | 19-Jul-02 | Georgia | Baker | USA | m | nr | a | Adam Miles, Steven Castelberry |
| AM-18 | 1,2 | KR337116 | KR362320, KR362321 | 02-Jun-02 | Georgia | Worth | USA | f | l | a | Adam Miles, Steven Castelberry |
| AM-19 | 1,2 | KR337117 | KR362322, KR362323 | 06-Jun-02 | Georgia | Worth | USA | f | l | a | Adam Miles, Steven Castelberry |
| AM-21 | 1,2 | KR337118 | KR362324, KR362325 | 17-Jun-02 | Georgia | Worth | USA | f | l | a | Adam Miles, Steven Castelberry |
| AM-23 | 1,2 |  | KR362326, KR362327 | 17-Jun-02 | Georgia | Worth | USA | f | l | a | Adam Miles, Steven Castelberry |
| AM-24 | 1,2 |  | KR362328, KR362329 | 17-Jun-02 | Georgia | Worth | USA | f | l | a | Adam Miles, Steven Castelberry |
| AM-38 | 1,2 | KR337119 | KR362330, KR362331 | 25-Jul-02 | Georgia | Worth | USA | m | nr | j | Adam Miles, Steven Castelberry |
| AM-40 | 1,2 |  |  | 25-Jul-02 | Georgia | Worth | USA | f | u | a | Adam Miles, Steven Castelberry |
| AM-41 | 1,2 | KR337120 | KR362332, KR362333 | 25-Jul-02 | Georgia | Worth | USA | m | nr | j | Adam Miles, Steven Castelberry |
| AM-42 | 1,2 |  |  | 25-Jul-02 | Georgia | Worth | USA | f | pl | a | Adam Miles, Steven Castelberry |
| AM-43 | 1,2 |  |  | 25-Jul-02 | Georgia | Worth | USA | f | pl | a | Adam Miles, Steven Castelberry |
| AM-46 | 1,2 |  |  | 30-Jul-02 | Georgia | Worth | USA | f | pl | a | Adam Miles, Steven Castelberry |
| AM-48 | 1,2 |  |  | 30-Jul-02 | Georgia | Worth | USA | f | pl | a | Adam Miles, Steven Castelberry |
| AM-50 | 1,2 |  |  | 30-Jul-02 | Georgia | Worth | USA | f | nr | a | Adam Miles, Steven Castelberry |
| AM-53 | 1,2 |  |  | 30-Jul-02 | Georgia | Worth | USA | f | nr | j | Adam Miles, Steven Castelberry |
| AM-56 | 1 | KR337121 | KR362334, KR362335 | 02-Jun-03 | Georgia | Worth | USA | f | l | a | Adam Miles, Steven Castelberry |
| AM-57 | 1 | KR337122 |  | 02-Jun-03 | Georgia | Worth | USA | f | p | a | Adam Miles, Steven Castelberry |
| AM-58 | 1 |  |  | 04-Jun-03 | Georgia | Worth | USA | f | l | a | Adam Miles, Steven Castelberry |
| AM-60 | 1 |  |  | 16-Jun-03 | Georgia | Worth | USA | f | l | a | Adam Miles, Steven Castelberry |
| AM-63 | 1 |  |  | 18-Jun-03 | Georgia | Worth | USA | f | l | a | Adam Miles, Steven Castelberry |
| AM-65 | 1 |  |  | 18-Jun-03 | Georgia | Worth | USA | f | l | a | Adam Miles, Steven Castelberry |
| AM-70 | 1 |  |  | 21-Jun-03 | Georgia | Worth | USA | f | l | a | Adam Miles, Steven Castelberry |
| AM-72 | 1 |  |  | 23-Jun-03 | Georgia | Worth | USA | f | l | a | Adam Miles, Steven Castelberry |
| AM-74 | 1 |  |  | 23-Jun-03 | Georgia | Worth | USA | f | l | a | Adam Miles, Steven Castelberry |
| AM-78 | 1 |  |  | 24-Jun-03 | Georgia | Worth | USA | f | l | a | Adam Miles, Steven Castelberry |
| AM-79 | 1 |  |  | 24-Jun-03 | Georgia | Worth | USA | f | l | a | Adam Miles, Steven Castelberry |
| AM-81 | 1 |  |  | 24-Jun-03 | Georgia | Worth | USA | m |  | j | Adam Miles, Steven Castelberry |
| AM-96 | 1 |  |  | 21-Jul-03 | Georgia | Worth | USA | m |  | j | Adam Miles, Steven Castelberry |
| AM-105 | 1 | KR337123 | KR362336, KR362337 | 24-Jul-02 | Georgia | Worth | USA | m |  | j | Adam Miles, Steven Castelberry |
| AM-114 | 1 | KR337124 | KR362338, KR362339 | 26-Jul-02 | Georgia | Worth | USA | m |  | j | Adam Miles, Steven Castelberry |
| IL-19 | 1 | KR337099 |  | 22-Jun-01 | Illinois | Union | USA | m | s | a | Tim Carter |
| IL-24 | 1 | KR337100 |  | 22-Jun-01 | Illinois | Union | USA | f | l | a | Tim Carter |
| IL-25 | 1 |  |  | 22-Jun-01 | Illinois | Union | USA | f | l | a | Tim Carter |
| IL-31 | 1 | KR337101 |  | 22-Jul-01 | Illinois | Jackson | USA | f |  | j | Tim Carter |
| IL-48 | 1 | KR337093 | KR362302, KR362303 | 30-May-00 | Illinois | Pope | USA | m |  | a | Tim Carter |
| IL-49 | 1 | KR337094 | KR362304, KR362305 | 30-May-00 | Illinois | Pope | USA | m |  | a | Tim Carter |
| IL-50 | 1 | KR337095 | KR362306, KR362307 | 30-May-00 | Illinois | Pope | USA | m |  | a | Tim Carter |
| IL-70 | 1 | KR337096 | KR362308, KR362309 | 06-Jun-00 | Illinois | Pope | USA | m |  | a | Tim Carter |
| IL-108 | 1 | KR337097 |  | 27-Jun-00 | Illinois | Williamson | USA | m |  | a | Tim Carter |
| IL-119 | 1 | KR337098 |  | 07-Jul-00 | Illinois | Jackson | USA | f | l | a | Tim Carter |
| IL-340 | 1 | KR337102 |  | 22-Jun-03 | Illinois | Union | USA | f | l | a | Tim Carter |
| IL-341 | 1 | KR337103 |  | 22-Jun-03 | Illinois | Union | USA | f | l | a | Tim Carter |
| IL-343 | 1 | KR337104 |  | 22-Jun-03 | Illinois | Union | USA | f | l | a | Tim Carter |
| IL-355 | 1 | KR337105 |  | 23-Jun-03 | Illinois | Union | USA | f | l | a | Tim Carter |
| IL-403 | 1 |  |  | 24-Jul-03 | Illinois | Pope | USA | f |  | j | Tim Carter |
| IL-404 | 1 | KR337106 | KR362312, KR362313 | 24-Jul-03 | Illinois | Pope | USA | f |  | j | Tim Carter |
| IL-405 | 1 | KR337107 | KR362314, KR362315 | 24-Jul-03 | Illinois | Pope | USA | f |  | j | Tim Carter |
| IL-406 | 1 | KR337108 | KR362316, KR362317 | 24-Jul-03 | Illinois | Pope | USA | m |  | j | Tim Carter |
| IL-407 | 1 | KR337109 | KR362318, KR362319 | 24-Jul-03 | Illinois | Pope | USA | m |  | j | Tim Carter |
| IL-408 | 1 | KR337110 |  | 24-Jul-03 | Illinois | Pope | USA | m |  | j | Tim Carter |
| IL-409 | 1 |  |  | 24-Jul-03 | Illinois | Pope | USA | f | l | a | Tim Carter |
| IL-410 | 1 | KR337111 |  | 24-Jul-03 | Illinois | Pope | USA | f |  | j | Tim Carter |
| IL-412 | 1 | KR337112 |  | 24-Jul-03 | Illinois | Pope | USA | m |  | a | Tim Carter |
| IL-415 | 1 |  |  | 24-Jul-03 | Illinois | Pope | USA | m |  | j | Tim Carter |
| IL-425 | 1 | KR337113 |  | 24-Jul-03 | Illinois | Pope | USA | f |  | j | Tim Carter |
| IL-426 | 1 | KR337114 |  | 24-Jul-03 | Illinois | Pope | USA | m |  | j | Tim Carter |
| JJ-01 | 1 | KR337185 | KR362390, KR362391 | 16-Jun-04 | Maryland | Washington | USA | f | l | a | Josh Johnson, Ed Gates |
| JJ-02 | 1 | KR337186 | KR362392, KR362393 | 17-Jun-04 | Maryland | Washington | USA | f | l | a | Josh Johnson, Ed Gates |
| JJ-10 | 1 |  |  | 29-Jun-04 | Maryland | Garrett | USA | m | nr | a | Josh Johnson, Ed Gates |
| JJ-22 | 1 | KR337187 | KR362394, KR362395 | 09-Jul-04 | Maryland | Allegany | USA | m | nr | a | Josh Johnson, Ed Gates |
| JJ-27 | 1 |  | KR362396, KR362397 | 09-Jul-04 | Maryland | Allegany | USA | m | nr | a | Josh Johnson, Ed Gates |
| JJ-39 | 1 | KR337188 | KR362398, KR362399 | 13-Jul-04 | Maryland | Frederick | USA | m | nr | a | Josh Johnson, Ed Gates |
| JJ-40 | 1 |  | KR362400, KR362401 | 13-Jul-04 | Maryland | Frederick | USA | m | s | a | Josh Johnson, Ed Gates |
| JJ-42 | 1 |  | KR362402, KR362403 | 13-Jul-04 | Maryland | Frederick | USA | f | l | a | Josh Johnson, Ed Gates |
| JJ-45 | 1 | KR337189 |  | 14-Jul-04 | Maryland | Frederick | USA | m | s | a | Josh Johnson, Ed Gates |
| JJ-57 | 1 |  |  | 14-Jul-04 | Maryland | Frederick | USA | m | s | a | Josh Johnson, Ed Gates |
| JJ-59 | 1 | KR337190 |  | 14-Jul-04 | Maryland | Frederick | USA | m | s | a | Josh Johnson, Ed Gates |
| JJ-62 | 1 | KR337191 |  | 15-Jul-04 | Maryland | Washington | USA | m | s | a | Josh Johnson, Ed Gates |
| JJ-66 | 1 |  | KR362404, KR362405 | 20-Jul-04 | Maryland | Allegany | USA | f | l | a | Josh Johnson, Ed Gates |
| JJ-67 | 1 | KR337192 |  | 20-Jul-04 | Maryland | Allegany | USA | m | nr | a | Josh Johnson, Ed Gates |
| JJ-69 | 1 | KR337193 |  | 21-Jul-04 | Maryland | Allegany | USA | f | nr | j | Josh Johnson, Ed Gates |
| JJ-78 | 1 |  |  | 05-Aug-04 | Maryland | Washington | USA | f | nr | j | Josh Johnson, Ed Gates |
| JJ-79 | 1 |  |  | 05-Aug-04 | Maryland | Washington | USA | m | nr | j | Josh Johnson, Ed Gates |
| JJ-82 | 1 |  |  | 06-Aug-04 | Maryland | Washington | USA | f | nr | j | Josh Johnson, Ed Gates |
| JJ-83 | 1 |  |  | 06-Aug-04 | Maryland | Washington | USA | f | nr | j | Josh Johnson, Ed Gates |
| JJ-99 | 1 |  |  | 09-Aug-04 | Maryland | Washington | USA | f | nr | a | Josh Johnson, Ed Gates |
| JJ-116 | 1 |  |  | 11-Aug-04 | Maryland | Washington | USA | m | s | a | Josh Johnson, Ed Gates |
| MV-19 | 1 |  | KR362412, KR362413 | 19-Jul-06 | Michigan | Allegan | USA | m |  | a | Maarten Vonhof, Ken Luzynski |
| MV-20 | 1 | KR337212 |  | 19-Jul-06 | Michigan | Allegan | USA | f | pl | a | Maarten Vonhof, Ken Luzynski |
| MV-21 | 1 |  |  | 19-Jul-06 | Michigan | Allegan | USA | m |  | a | Maarten Vonhof, Ken Luzynski |
| MV-22 | 1 | KR337213 | KR362414, KR362415 | 22-Jul-06 | Michigan | Allegan | USA | m |  | a | Ken Luzynski |
| MV-55 | 1 |  |  | 26-Jul-06 | Michigan | Allegan | USA | m |  | a | Ken Luzynski |
| LW-07 | 1 | KR337206 |  | 28-Jul-05 | Michigan | Barry | USA | f |  | j | Lisa Winhold |
| LW-08 | 1 | KR337207 |  | 28-Jul-05 | Michigan | Barry | USA | m |  | a | Lisa Winhold |
| LW-06 | 1 |  |  | 27-Jul-05 | Michigan | Berrien | USA | f |  | j | Lisa Winhold |
| LW-05 | 1 | KR337205 |  | 24-Jul-05 | Michigan | Calhoun | USA | m |  | j | Lisa Winhold |
| LW-18 | 1 |  |  | 21-Jun-05 | Michigan | Cass | USA | f |  | a | Lisa Winhold |
| LW-19 | 1 |  |  | 25-Jun-05 | Michigan | Cass | USA | f |  | a | Lisa Winhold |
| LW-20 | 1 |  | KR362408, KR362409 | 25-Jun-05 | Michigan | Cass | USA | m |  | a | Lisa Winhold |
| LW-03 | 1 | KR337203 |  | 23-Jul-05 | Michigan | St. Joseph | USA | m |  | a | Lisa Winhold |
| LW-04 | 1 | KR337204 |  | 23-Jul-05 | Michigan | St. Joseph | USA | f |  | j | Lisa Winhold |
| LW-17 | 1 |  | KR362406, KR362407 | 09-Jun-05 | Michigan | St. Joseph | USA | m |  | a | Lisa Winhold |
| LW-21 | 1 |  | KR362410, KR362411 | 06-Jul-05 | Michigan | St. Joseph | USA | f |  | a | Lisa Winhold |
| LW-22 | 1 | KR337208 |  | 06-Jul-05 | Michigan | St. Joseph | USA | m |  | a | Lisa Winhold |
| SA-89 | 1,2 |  |  | 19-Jun-02 | Missouri | Texas | USA | f | l | a | Sybil Amelon |
| SA-90 | 1,2 |  |  | 19-Jun-02 | Missouri | Texas | USA | f | l | a | Sybil Amelon |
| SA-91 | 1,2 |  |  | 19-Jun-02 | Missouri | Texas | USA | f | l | a | Sybil Amelon |
| SA-92 | 1,2 |  |  | 19-Jun-02 | Missouri | Texas | USA | m | ns | a | Sybil Amelon |
| SA-93 | 1,2 |  | KR362454, KR362455 | 20-Jun-02 | Missouri | Texas | USA | f | l | a | Sybil Amelon |
| SA-94 | 1,2 |  |  | 20-Jun-02 | Missouri | Texas | USA | m | ns | a | Sybil Amelon |
| SA-95 | 1,2 | KR337246 |  | 21-Jun-02 | Missouri | Texas | USA | f | l | a | Sybil Amelon |
| SA-96 | 1,2 | KR337247 |  | 21-Jun-02 | Missouri | Texas | USA | f | l | a | Sybil Amelon |
| SA-98 | 1,2 | KR337248 | KR362456, KR362457 | 22-Jun-02 | Missouri | Texas | USA | m | ns | a | Sybil Amelon |
| SA-99 | 1,2 |  |  | 22-Jun-02 | Missouri | Texas | USA | f | l | a | Sybil Amelon |
| SA-101 | 1,2 | KR337249 |  | 22-Jun-02 | Missouri | Texas | USA | f | l | a | Sybil Amelon |
| SA-102 | 1,2 |  |  | 22-Jun-02 | Missouri | Texas | USA | f | l | a | Sybil Amelon |
| SA-103 | 1,2 |  |  | 22-Jun-02 | Missouri | Texas | USA | f | l | a | Sybil Amelon |
| SA-114 | 1,2 |  | KR362458, KR362459 | 08-Jul-02 | Missouri | Texas | USA | f | l | a | Sybil Amelon |
| SA-115 | 1,2 |  |  | 09-Jul-02 | Missouri | Texas | USA | m | ns | a | Sybil Amelon |
| SA-116 | 1,2 |  |  | 09-Jul-02 | Missouri | Texas | USA | f | l | a | Sybil Amelon |
| SA-121 | 1,2 |  | KR362460, KR362461 | 10-Jul-02 | Missouri | Texas | USA | f | pl | a | Sybil Amelon |
| SA-122 | 1,2 | KR337250 | KR362462, KR362463 | 11-Jul-02 | Missouri | Texas | USA | f | nr | a | Sybil Amelon |
| SA-123 | 1,2 |  | KR362464, KR362465 | 14-Jul-02 | Missouri | Texas | USA | f | nr | a | Sybil Amelon |
| SA-124 | 1,2 |  | KR362466, KR362467 | 14-Jul-02 | Missouri | Texas | USA | f | pl | a | Sybil Amelon |
| SA-126 | 1,2 |  | KR362468, KR362469 | 14-Jul-02 | Missouri | Texas | USA | m | ns | a | Sybil Amelon |
| SA-127 | 1,2 | KR337251 | KR362470, KR362471 | 14-Jul-02 | Missouri | Texas | USA | m | ns | a | Sybil Amelon |
| SA-128 | 1,2 |  |  | 14-Jul-02 | Missouri | Texas | USA | f | pl | a | Sybil Amelon |
| SA-129 | 1,2 | KR337252 |  | 14-Jul-02 | Missouri | Texas | USA | f | nr | a | Sybil Amelon |
| SA-133 | 1,2 | KR337253 |  | 18-Jul-02 | Missouri | Texas | USA | f | nr | a | Sybil Amelon |
| SA-138 | 1,2 |  |  | 19-Jul-02 | Missouri | Texas | USA | f | l | a | Sybil Amelon |
| SA-139 | 1,2 |  | KR362472, KR362473 | 19-Jul-02 | Missouri | Texas | USA | f | l | a | Sybil Amelon |
| EB-314 | 1 | KR337173 |  | 08-Jun-05 | North Carolina | Cumberland | USA | f |  | a | Eric Britzke |
| EB-315 | 1 | KR337174 | KR362386, KR362387 | 08-Jun-05 | North Carolina | Cumberland | USA | f |  | a | Eric Britzke |
| EB-316 | 1 | KR337175 |  | 08-Jun-05 | North Carolina | Cumberland | USA | f |  | a | Eric Britzke |
| EB-317 | 1 |  |  | 12-Jun-05 | North Carolina | Cumberland | USA | m |  | a | Eric Britzke |
| GL-01 | 1 |  | KR362388, KR362389 | 11-Jul-01 | North Carolina | Graham | USA | f |  | a | Garry Libby |
| GL-02 | 1 | KR337181 |  | 11-Jul-01 | North Carolina | Graham | USA | f | l | a | Garry Libby |
| GL-03 | 1 |  |  | 11-Jul-01 | North Carolina | Graham | USA | m |  | a | Garry Libby |
| GL-04 | 1 | KR337182 |  | 12-Jul-01 | North Carolina | Graham | USA | m |  | a | Garry Libby |
| GL-08 | 1 | KR337183 |  | 12-Jul-01 | North Carolina | Graham | USA | m |  | a | Garry Libby |
| MG-49 | 1 |  |  | 12-Jun-06 | North Carolina | Hoke | USA | m |  | a | Mark Gumbert |
| SC-01 | 1 | KR337254 |  | 15-Jun-06 | North Carolina | Rutherford | USA | m |  | a | Jeff Schwierjohann |
| SC-02 | 1 |  |  | 15-Jun-06 | North Carolina | Rutherford | USA | m |  | a | Jeff Schwierjohann |
| SC-03 | 1 |  |  | 15-Jun-06 | North Carolina | Rutherford | USA | m |  | a | Jeff Schwierjohann |
| NC-01 | 1 | KR337214 |  | 07-Jun-01 | North Carolina | Stokes | USA | f | p | a | Mary Kay Clark, Seth Lambiase |
| NC-02 | 1 |  |  | 07-Jun-01 | North Carolina | Stokes | USA | m | nr | a | Mary Kay Clark, Seth Lambiase |
| NC-03 | 1 | KR337215 |  | 08-Jun-01 | North Carolina | Stokes | USA | f | l | a | Mary Kay Clark, Seth Lambiase |
| NC-04 | 1 |  |  | 08-Jun-01 | North Carolina | Stokes | USA | f | p | a | Mary Kay Clark, Seth Lambiase |
| EB-318 | 1 |  |  | 12-Jun-05 | North Carolina |  | USA | m |  | j | Eric Britzke |
| ON-01 | 1,2 | KR337218 | KR362416, KR362417 | 24-Jun-05 | Ontario |  | Canada |  |  |  | Liz Reddy |
| ON-02 | 1,2 | KR337219 | KR362418, KR362419 | 24-Jun-05 | Ontario |  | Canada |  |  |  | Liz Reddy |
| ON-03 | 1,2 |  | KR362420, KR362421 | 24-Jun-05 | Ontario |  | Canada |  |  |  | Liz Reddy |
| ON-04 | 1,2 |  | KR362422, KR362423 | 24-Jun-05 | Ontario |  | Canada |  |  |  | Liz Reddy |
| ON-05 | 1,2 | KR337220 | KR362424, KR362425 | 24-Jun-05 | Ontario |  | Canada |  |  |  | Liz Reddy |
| ON-06 | 1,2 | KR337221 | KR362426, KR362427 | 24-Jun-05 | Ontario |  | Canada |  |  |  | Liz Reddy |
| ON-07 | 1,2 |  | KR362428, KR362429 | 24-Jun-05 | Ontario |  | Canada |  |  |  | Liz Reddy |
| ON-08 | 1,2 |  | KR362430, KR362431 | 24-Jun-05 | Ontario |  | Canada |  |  |  | Liz Reddy |
| ON-09 | 1,2 | KR337222 | KR362432, KR362433 | 24-Jun-05 | Ontario |  | Canada |  |  |  | Liz Reddy |
| ON-10 | 1,2 | KR337223 | KR362434, KR362435 | 24-Jun-05 | Ontario |  | Canada |  |  |  | Liz Reddy |
| ON-11 | 1,2 | KR337224 |  | 24-Jun-05 | Ontario |  | Canada |  |  |  | Liz Reddy |
| ON-12 | 1,2 |  |  | 24-Jun-05 | Ontario |  | Canada |  |  |  | Liz Reddy |
| ON-13 | 1,2 |  |  | 24-Jun-05 | Ontario |  | Canada |  |  |  | Liz Reddy |
| ON-14 | 1,2 | KR337225 |  | 24-Jun-05 | Ontario |  | Canada |  |  |  | Liz Reddy |
| ON-15 | 1,2 |  |  | 24-Jun-05 | Ontario |  | Canada |  |  |  | Liz Reddy |
| ON-16 | 1,2 |  |  | 24-Jun-05 | Ontario |  | Canada |  |  |  | Liz Reddy |
| ON-17 | 1,2 | KR337226 |  | 24-Jun-05 | Ontario |  | Canada |  |  |  | Liz Reddy |
| ON-18 | 1,2 | KR337227 |  | 24-Jun-05 | Ontario |  | Canada |  |  |  | Liz Reddy |
| ON-19 | 1,2 |  |  | 24-Jun-05 | Ontario |  | Canada |  |  |  | Liz Reddy |
| BMW-124 | 1 | KR337156 |  | 17-Jul-03 | Tennessee | Anderson | USA | m |  | a | Jenny Fiedler |
| EB-83 | 1,2 |  | KR362378, KR362379 | 18-Jun-02 | Tennessee | Blount | USA | f | l | a | Eric Britzke |
| EB-84 | 1,2 | KR337167 | KR362380, KR362381 | 18-Jun-02 | Tennessee | Blount | USA | m | nr | a | Eric Britzke |
| EB-85 | 1,2 | KR337168 | KR362382, KR362383 | 18-Jun-02 | Tennessee | Blount | USA | m | nr | a | Eric Britzke |
| EB-86 | 1,2 | KR337169 | KR362384, KR362385 | 18-Jun-02 | Tennessee | Blount | USA | m | nr | a | Eric Britzke |
| SMNP-101 | 1,2 |  | KR362476, KR362477 | 17-Jun-02 | Tennessee | Blount | USA | m |  | a | Bat Blitz People |
| SMNP-103 | 1,2 | KR337255 |  | 17-Jun-02 | Tennessee | Blount | USA | m | nr | a | Bat Blitz People |
| SMNP-98 | 1,2 |  | KR362474, KR362475 | 17-Jun-02 | Tennessee | Blount | USA | m |  | a | Bat Blitz People |
| MG-47 | 1 | KR337209 |  | 29-Jul-06 | Tennessee | Campbell | USA | m |  | j | Mark Gumbert |
| MG-48 | 1 | KR337210 |  | 29-Jul-06 | Tennessee | Campbell | USA | f |  | a | Mark Gumbert |
| MG-51 | 1 |  |  | 01-Jun-06 | Tennessee | Campbell | USA | m |  | a | Mark Gumbert |
| MG-50 | 1 | KR337211 |  | 10-Jul-06 | Tennessee | Cocke | USA | m |  | j | Mark Gumbert |
| MG-52 | 1 |  |  | 12-Jul-06 | Tennessee | Cocke | USA | m |  | j | Mark Gumbert |
| TN-35 | 1 | KR337256 |  | 18-Jul-00 | Tennessee | Hamblen | USA | m |  | a | Tennessee Rabies Lab |
| TN-769 | 1,2 |  |  | 25-Jun-02 | Tennessee | Hamilton | USA | f | l | a | Tennessee Rabies Lab |
| MG-41 | 1 |  |  | 19-Jul-06 | Tennessee | Monroe | USA | m |  | j | Mark Gumbert |
| MG-42 | 1 |  |  | 19-Jul-06 | Tennessee | Monroe | USA | m |  | a | Mark Gumbert |
| MG-43 | 1 |  |  | 19-Jul-06 | Tennessee | Monroe | USA | m |  | j | Mark Gumbert |
| TN-803 | 1 |  |  | 22-Jun-01 | Tennessee | Sullivan | USA | f | l | a | Tennessee Rabies Lab |
| TN-804 | 1 |  |  | 22-Jun-01 | Tennessee | Sullivan | USA | m |  | j | Tennessee Rabies Lab |
| TN-806 | 1 |  |  | 22-Jun-01 | Tennessee | Sullivan | USA | f |  | j | Tennessee Rabies Lab |
| TN-797 | 1 | KR337257 |  | 21-Jun-01 | Tennessee | Washington | USA | m |  | a | Tennessee Rabies Lab |
| ASK1081 | 1 | KR337126 | KR362340, KR362341 | 04-May-86 | Texas | Tom Green | USA | f |  |  | Angelo State Natural History Collection |
| ASK1084 | 1 | KR337127 | KR362342, KR362343 | 11-May-86 | Texas | Tom Green | USA | f |  |  | Angelo State Natural History Collection |
| ASK1085 | 1 |  | KR362344, KR362345 | 11-May-86 | Texas | Tom Green | USA | f |  |  | Angelo State Natural History Collection |
| ASK1086 | 1 | KR337128 | KR362346, KR362347 | 11-May-86 | Texas | Tom Green | USA | f |  |  | Angelo State Natural History Collection |
| ASK3698 | 1 | KR337129 |  | 11-Jul-92 | Texas | Tom Green | USA | f |  |  | Angelo State Natural History Collection |
| ASK3711 | 1 | KR337130 | KR362348, KR362349 | 13-Jul-92 | Texas | Tom Green | USA | f |  |  | Angelo State Natural History Collection |
| ASK3716 | 1 | KR337131 | KR362350, KR362351 | 11-Jul-92 | Texas | Tom Green | USA | f |  |  | Angelo State Natural History Collection |
| ASK3730 | 1 | KR337132 | KR362352, KR362353 | 24-Aug-92 | Texas | Tom Green | USA | f |  |  | Angelo State Natural History Collection |
| ASK3739 | 1 | KR337133 | KR362354, KR362355 | 22-May-92 | Texas | Tom Green | USA | f |  |  | Angelo State Natural History Collection |
| ASK3740 | 1 | KR337134 |  | 22-May-92 | Texas | Tom Green | USA | f |  |  | Angelo State Natural History Collection |
| ASK3741 | 1 | KR337135 |  | 22-May-92 | Texas | Tom Green | USA | f |  |  | Angelo State Natural History Collection |
| ASK3742 | 1 | KR337136 |  | 22-May-92 | Texas | Tom Green | USA | f |  |  | Angelo State Natural History Collection |
| ASK3746 | 1 | KR337137 |  | 22-May-92 | Texas | Tom Green | USA | f |  |  | Angelo State Natural History Collection |
| ASK4351 | 1 | KR337138 |  | 27-Apr-01 | Texas | Tom Green | USA | f |  |  | Angelo State Natural History Collection |
| ASK4475 | 1 | KR337139 |  | 07-Jul-00 | Texas | Tom Green | USA | f |  |  | Angelo State Natural History Collection |
| ASK4478 | 1 | KR337140 |  | 06-Jul-00 | Texas | Tom Green | USA | f |  |  | Angelo State Natural History Collection |
| ASK4489 | 1 |  |  | 22-Jun-00 | Texas | Tom Green | USA | f |  |  | Angelo State Natural History Collection |
| ASK4490 | 1 |  |  | 23-Jun-00 | Texas | Tom Green | USA | f |  |  | Angelo State Natural History Collection |
| ASK5103 | 1 |  |  | 31-Jul-00 | Texas | Tom Green | USA | f |  |  | Angelo State Natural History Collection |
| ASK5188 | 1 | KR337141 |  | 26-Jun-00 | Texas | Tom Green | USA | f |  |  | Angelo State Natural History Collection |
| ASK6059 | 2 | KR337143 |  | 24-Aug-02 | Texas | Brown | USA | f |  |  | Angelo State Natural History Collection |
| CS-135 | 1 | KR337165 |  | 24-Jun-05 | West Virginia | Hardy | USA | u |  | u | Craig Stihler |
| EB-319 | 1 | KR337176 |  | 17-Jun-05 | West Virginia | Hardy | USA | f |  | a | Eric Britzke |
| EB-320 | 1 |  |  | 17-Jun-05 | West Virginia | Hardy | USA | m |  | j | Eric Britzke |
| EB-321 | 1 | KR337177 |  | 19-Jun-05 | West Virginia | Hardy | USA | m |  | a | Eric Britzke |
| EB-322 | 1 |  |  | 22-Jun-05 | West Virginia | Hardy | USA | f |  | a | Eric Britzke |
| EB-323 | 1 | KR337178 |  | 22-Jun-05 | West Virginia | Hardy | USA | f |  | a | Eric Britzke |
| EB-324 | 1 | KR337179 |  | 22-Jun-05 | West Virginia | Hardy | USA | f |  | a | Eric Britzke |
| EB-325 | 1 |  |  | 22-Jun-05 | West Virginia | Hardy | USA | f |  | a | Eric Britzke |
| CS-88 | 1 |  |  | 29-Jul-03 | West Virginia | Pendleton | USA | m |  | j | Craig Stihler |
| CS-92 | 1 | KR337158 | KR362360, KR362361 | 29-Jul-03 | West Virginia | Pendleton | USA | m | nr | a | Craig Stihler |
| CS-96 | 1 | KR337159 | KR362362, KR362363 | 30-Jul-03 | West Virginia | Pendleton | USA | m | nr | a | Craig Stihler |
| CS-101 | 1 | KR337160 |  | 30-Jul-03 | West Virginia | Pendleton | USA | m | nr | a | Craig Stihler |
| CS-111 | 1 |  | KR362364, KR362365 | 30-Jul-03 | West Virginia | Pendleton | USA | m | nr | a | Craig Stihler |
| CS-113 | 1 |  | KR362366, KR362367 | 30-Jul-03 | West Virginia | Pendleton | USA | m | nr | a | Craig Stihler |
| CS-117 | 1 |  | KR362368, KR362369 | 29-Jul-03 | West Virginia | Pendleton | USA | f | nr | a | Craig Stihler |
| CS-118 | 1 |  | KR362370, KR362371 | 29-Jul-03 | West Virginia | Pendleton | USA | m | nr | a | Craig Stihler |
| CS-119 | 1 | KR337161 | KR362372, KR362373 | 30-Jul-03 | West Virginia | Pendleton | USA | m | s | a | Craig Stihler |
| CS-121 | 1 | KR337162 | KR362374, KR362375 | 30-Jul-03 | West Virginia | Pendleton | USA | m | nr | a | Craig Stihler |
| CS-132 | 1 | KR337163 | KR362376, KR362377 | 31-Jul-03 | West Virginia | Pendleton | USA | m | s | a | Craig Stihler |
| CS-133 | 1 | KR337164 |  | 30-Jul-03 | West Virginia | Pendleton | USA | m |  | j | Craig Stihler |
| CS-50 | 1,2 |  |  | 30-Jul-02 | West Virginia | Mason | USA | m |  | j | Craig Stihler |
| CS-51 | 1,2 |  |  | 30-Jul-02 | West Virginia | Mason | USA | m |  | j | Craig Stihler |
| CS-52 | 1,2 |  |  | 30-Jul-02 | West Virginia | Mason | USA | m |  | j | Craig Stihler |
| CS-53 | 1,2 |  |  | 30-Jul-02 | West Virginia | Mason | USA | m |  | j | Craig Stihler |
| CS-54 | 1,2 |  |  | 30-Jul-02 | West Virginia | Mason | USA | f |  | j | Craig Stihler |
| CS-55 | 1,2 |  |  | 30-Jul-02 | West Virginia | Mason | USA | m |  | j | Craig Stihler |
| CS-56 | 1,2 |  |  | 30-Jul-02 | West Virginia | Mason | USA | f |  | j | Craig Stihler |
| CS-57 | 1,2 |  |  | 31-Jul-02 | West Virginia | Mason | USA | m |  | j | Craig Stihler |
| CS-58 | 1,2 |  |  | 31-Jul-02 | West Virginia | Mason | USA | m | nr | a | Craig Stihler |
| CS-59 | 1,2 |  |  | 31-Jul-02 | West Virginia | Mason | USA | f | pl | a | Craig Stihler |
| CS-60 | 1,2 |  |  | 31-Jul-02 | West Virginia | Mason | USA | f |  | j | Craig Stihler |
| CS-61 | 1,2 |  |  | 31-Jul-02 | West Virginia | Mason | USA | f | nr | a | Craig Stihler |
| CS-62 | 1,2 |  |  | 31-Jul-02 | West Virginia | Mason | USA | m | s | a | Craig Stihler |
| CS-63 | 1,2 |  |  | 31-Jul-02 | West Virginia | Mason | USA | m | s | a | Craig Stihler |
| CS-64 | 1,2 |  |  | 31-Jul-02 | West Virginia | Mason | USA | m | s | a | Craig Stihler |
| CS-65 | 1,2 |  |  | 31-Jul-02 | West Virginia | Mason | USA | f | pl | a | Craig Stihler |
| CS-66 | 1,2 |  |  | 31-Jul-02 | West Virginia | Mason | USA | f | pl | a | Craig Stihler |
| CS-67 | 1,2 |  |  | 31-Jul-02 | West Virginia | Mason | USA | m | s | a | Craig Stihler |
| CS-68 | 1,2 |  |  | 31-Jul-02 | West Virginia | Mason | USA | m | s | a | Craig Stihler |
| CS-69 | 1,2 |  |  | 31-Jul-02 | West Virginia | Mason | USA | m | s | a | Craig Stihler |
| CS-70 | 1,2 |  |  | 31-Jul-02 | West Virginia | Mason | USA | f |  | j | Craig Stihler |
| CS-71 | 1,2 |  |  | 31-Jul-02 | West Virginia | Mason | USA | m |  | j | Craig Stihler |
| CS-72 | 1,2 |  |  | 31-Jul-02 | West Virginia | Mason | USA | m |  | j | Craig Stihler |
| CS-73 | 1,2 |  |  | 31-Jul-02 | West Virginia | Mason | USA | m |  | j | Craig Stihler |
| CS-74 | 1,2 |  |  | 31-Jul-02 | West Virginia | Mason | USA | m |  | j | Craig Stihler |
| CM-102846 | 1 | KR337091 | KR362358, KR362359 | 29-Jul-87 | West Virginia | Fayette | USA | m |  |  | Carnegie Museum of Natural History |
| M-6260 | 1 | KR337092 |  |  | Louisiana |  | USA |  |  |  | Louisiana Museum of Natural History |
| IL-43 | 1 |  | KR362310, KR362311 | 05-Sep-02 | Illinois | Wayne | USA | m |  | a | Tim Carter |
| AR-110 | 1 | KR337125 |  | 13-Jul-04 | Texas | Cass | USA | m |  | j | David Saugey |
| AR-111 | 1 |  |  | 13-Jul-04 | Texas | Cass | USA | m |  | j | David Saugey |
| ASK6012 | 1,2 | KR337142 |  | 13-Jun-02 | Louisiana | Winn | USA | f |  |  | Angelo State Natural History Collection |
| BL-09 | 1 | KR337144 |  | 04-Aug-03 | Arkansas | Montgomery | USA | f |  | a | David Saugey |
| BL-14 | 1 | KR337145 |  | 05-Aug-03 | Arkansas | Montgomery | USA | f |  | a | David Saugey |
| BL-20 | 1 | KR337146 |  | 05-Aug-03 | Arkansas | Montgomery | USA | f |  | a | David Saugey |
| BL-24 | 1 | KR337147 |  | 05-Aug-03 | Arkansas | Montgomery | USA | f |  | a | David Saugey |
| BL-32 | 1 | KR337148 |  | 05-Aug-03 | Arkansas | Montgomery | USA | f |  | a | David Saugey |
| BL-34 | 1 | KR337149 |  | 05-Aug-03 | Arkansas | Montgomery | USA | f |  | a | David Saugey |
| BL-66 | 1 |  |  | 06-Aug-03 | Arkansas | Yell | USA | f |  | a | David Saugey |
| BL-70 | 1 | KR337150 |  | 06-Aug-03 | Arkansas | Yell | USA | f |  | a | David Saugey |
| BL-74 | 1 | KR337151 |  | 06-Aug-03 | Arkansas | Yell | USA | f |  | a | David Saugey |
| BMW-20 | 1 | KR337152 | KR362356, KR362357 | 11-Jul-01 | Tennessee | Anderson | USA | m |  | a | Jenny Fiedler |
| BMW-36 | 1,2 | KR337153 |  | 11-Jun-02 | Tennessee | Anderson | USA | m |  | a | Jenny Fiedler |
| BMW-39 | 1,2 | KR337154 |  | 11-Jun-02 | Tennessee | Anderson | USA | m |  | a | Jenny Fiedler |
| BMW-41 | 1,2 |  |  | 25-Jun-02 | Tennessee | Anderson | USA | m |  | a | Jenny Fiedler |
| BMW-42 | 1,2 | KR337155 |  | 25-Jun-02 | Tennessee | Anderson | USA | m |  | a | Jenny Fiedler |
| CB-32 | 1 |  |  | 31-Jul-05 | Pennsylvania | Lebanon | USA | m |  | a | Cal Butchkoski |
| CH-01 | 1 | KR337157 |  | 24-Jul-01 | Saskatchewan |  | Canada | f |  | a | Craig Willis |
| DS-04 | 1 | KR337166 |  | 16-Jul-01 | Indiana | Hendriks | USA | m |  | j | Dale Sparks |
| EB-97 | 1,2 | KR337170 |  | 21-Jun-02 | Kentucky | Rowan | USA | f | l | a | Eric Britzke |
| EB-124 | 1,2 |  |  | 24-Jun-02 | Kentucky | Rowan | USA | m | nr | a | Eric Britzke |
| EB-126 | 1,2 | KR337171 |  | 24-Jun-02 | Kentucky | Rowan | USA | m | nr | a | Eric Britzke |
| EB-129 | 1,2 | KR337172 |  | 24-Jun-02 | Kentucky | Rowan | USA | m | nr | a | Eric Britzke |
| EB-163 | 1,2 |  |  | 18-Jul-02 | Kentucky | Rowan | USA | f | pl | a | Eric Britzke |
| EB-319 | 1 |  |  | 17-Jun-05 | West Virginia | Hardy | USA | f |  | a | Eric Britzke |
| EB-321 | 1 |  |  | 19-Jun-05 | West Virginia | Hardy | USA | m |  | a | Eric Britzke |
| EB-322 | 1 |  |  | 22-Jun-05 | West Virginia | Hardy | USA | f |  | a | Eric Britzke |
| EB-323 | 1 |  |  | 22-Jun-05 | West Virginia | Hardy | USA | f |  | a | Eric Britzke |
| EB-324 | 1 |  |  | 22-Jun-05 | West Virginia | Hardy | USA | f |  | a | Eric Britzke |
| EB-325 | 1 |  |  | 22-Jun-05 | West Virginia | Hardy | USA | f |  | a | Eric Britzke |
| FMSP-03 | 1,2 | KR337180 |  | 17-Jul-02 | North Carolina | Carteret | USA | f | nr | a | Seth Lambiase |
| HH-21 | 1 |  |  | 03-Aug-07 | Ontario |  | Canada | m |  | j | Hannah ter Hofstede |
| HR-08 | 1 |  |  | 22-Jul-01 | Michigan | Leelanau | USA | m |  | a | Heidi Rice |
| HR-10 | 1 | KR337184 |  | 22-Jul-01 | Michigan | Leelanau | USA | m |  | a | Heidi Rice |
| JK-29 | 1 | KR337194 |  | 17-Jul-05 | Kentucky | Trigg | USA | m |  | j | James Kiser |
| JK-30 | 1 |  |  | 17-Jul-05 | Kentucky | Trigg | USA | f |  | j | James Kiser |
| JK-31 | 1 | KR337195 |  | 17-Jul-05 | Kentucky | Trigg | USA | f |  | j | James Kiser |
| JK-32 | 1 |  |  | 17-Jul-05 | Kentucky | Trigg | USA | f |  | j | James Kiser |
| JK-33 | 1 |  |  | 17-Jul-05 | Kentucky | Trigg | USA | f |  | a | James Kiser |
| JK-34 | 1 | KR337196 |  | 18-Jul-05 | Kentucky | Trigg | USA | f |  | a | James Kiser |
| JK-35 | 1 |  |  | 18-Jul-05 | Kentucky | Trigg | USA | f |  | j | James Kiser |
| JK-36 | 1 |  |  | 18-Jul-05 | Kentucky | Trigg | USA | f |  | j | James Kiser |
| Lb-1X | 1 | KR337197 |  | 07-Jun-01 | Texas | Travis | USA | m |  | j | Barbara French |
| Lb-04 | 1 | KR337198 |  | 07-Jun-01 | Texas | Travis | USA | m |  | j | Barbara French |
| Lb-07 | 1 | KR337199 |  | 09-Jun-01 | Texas | Travis | USA | m |  | j | Barbara French |
| Lb-13 | 1 | KR337200 |  | 11-Jun-01 | Texas | Bexar | USA | f |  | j | Barbara French |
| Lb-14 | 1 |  |  | 11-Jun-01 | Texas | Bexar | USA | m |  | j | Barbara French |
| Lb-15 | 1 | KR337201 |  | 11-Jun-01 | Texas | Bexar | USA | f |  | j | Barbara French |
| Lb-16 | 1 |  |  | 11-Jun-01 | Texas | Bexar | USA | m |  | j | Barbara French |
| Lb-17 | 1 |  |  | 11-Jun-01 | Texas | Bexar | USA | f |  | j | Barbara French |
| Lb-22 | 1 | KR337202 |  | 12-Jun-01 | Texas | Travis | USA | f |  | a | Barbara French |
| Lb-23 | 1 |  |  | 12-Jun-01 | Texas | Travis | USA | f |  | j | Barbara French |
| Lb-24 | 1 |  |  | 12-Jun-01 | Texas | Travis | USA | f |  | j | Barbara French |
| Lb-25 | 1 |  |  | 12-Jun-01 | Texas | Travis | USA | f |  | j | Barbara French |
| Lb-26 | 1 |  |  | 12-Jun-01 | Texas | Travis | USA | m |  | j | Barbara French |
| Lb-27 | 1 |  |  | 11-Jun-01 | Texas | Travis | USA | f |  | a | Barbara French |
| Lb-28 | 1 |  |  | 11-Jun-01 | Texas | Travis | USA | m |  | j | Barbara French |
| Lb-29 | 1 |  |  | 11-Jun-01 | Texas | Travis | USA | f |  | j | Barbara French |
| MC-47 | 1 |  |  | 08-Aug-07 | New Jersey | Somerset | USA |  |  |  | Noelle Ronan |
| NYSDOH-01 | 1 | KR337216 |  | 17-Jun-05 | New York | Ulster | USA | m |  | a | NY State Dept. of Health |
| NYSDOH-03 | 1 | KR337217 |  | 10-Jul-04 | New York | Suffolk | USA | m |  | j | NY State Dept. of Health |
| RB-5845 | 1 |  | KR362436, KR362437 | 02-Aug-04 | Nebraska | Dawes | USA | m |  | a | Russ Benedict |
| RB-5864 | 1 |  |  | 09-Aug-04 | Nebraska | Holt | USA | f |  | j | Russ Benedict |
| RB-5870 | 1 |  |  | 10-Aug-04 | Nebraska | Knox | USA | m |  | a | Russ Benedict |
| SA-69 | 1,2 | KR337242 |  | 06-Jun-02 | Missouri | Carter | USA | f | l | a | Sybil Amelon |
| SA-71 | 1,2 | KR337243 |  | 06-Jun-02 | Missouri | Carter | USA | f | l | a | Sybil Amelon |
| SA-73 | 1,2 |  |  | 06-Jun-02 | Missouri | Carter | USA | m | ns | a | Sybil Amelon |
| SA-77 | 1,2 | KR337244 |  | 07-Jun-02 | Missouri | Carter | USA | f | l | a | Sybil Amelon |
| SA-79 | 1,2 |  |  | 07-Jun-02 | Missouri | Carter | USA | m | ns | a | Sybil Amelon |
| SA-80 | 1,2 | KR337245 |  | 07-Jun-02 | Missouri | Carter | USA | m | ns | a | Sybil Amelon |
| SA-83 | 1,2 |  |  | 07-Jun-02 | Missouri | Carter | USA | f | l | a | Sybil Amelon |
| SA-84 | 1,2 |  |  | 13-Jun-02 | Missouri | Carter | USA | f | l | a | Sybil Amelon |
| SA-88 | 1,2 |  |  | 13-Jun-02 | Missouri | Carter | USA | f | l | a | Sybil Amelon |
| SR-03 | 1 |  |  | 12-Jun-05 | West Virginia | Pocahontas | USA | f |  | a | Chris Sanders |
| VS-19 | 1,2 |  |  | 10-Jul-02 | South Dakota | Minnehaha | USA | f |  | j | Vicki Swier |
| VS-15 | 1,2 |  |  | 28-Jul-02 | South Dakota | Potter | USA | m |  | j | Vicki Swier |
| VS-16 | 1,2 |  |  | 28-Jul-02 | South Dakota | Potter | USA | f |  | j | Vicki Swier |
| VT-09 | 1 |  |  | 25-Jul-06 | Vermont | Addison | USA | m |  | a | Scott Darling |
| RP-62 | 2 |  |  | 29-Jul-02 | Arkansas | Saline | USA | m |  | j | Roger Perry |
| RP-63 | 2 |  |  | 29-Jul-02 | Arkansas | Saline | USA | m |  | j | Roger Perry |
| RP-64 | 2 |  |  | 29-Aug-02 | Arkansas | Saline | USA | f |  | a | Roger Perry |
| RP-65 | 2 |  |  | 29-Aug-02 | Arkansas | Saline | USA | m | s | a | Roger Perry |
| RP-66 | 2 |  |  | 29-Aug-02 | Arkansas | Saline | USA | f |  | a | Roger Perry |
| RP-67 | 2 |  |  | 29-Aug-02 | Arkansas | Saline | USA | m | s | a | Roger Perry |
| RP-68 | 2 |  |  | 29-Aug-02 | Arkansas | Saline | USA | m | s | a | Roger Perry |
| LF-08 | 2 |  |  | 17-May-02 | Florida | Orange | USA | f |  | j | Laura Finn |
| AM-12 | 2 |  |  | 28-May-02 | Georgia | Worth | USA | f | p | a | Adam Miles, Steven Castelberry |
| AM-13 | 2 |  |  | 28-May-02 | Georgia | Worth | USA | f | p | a | Adam Miles, Steven Castelberry |
| AM-14 | 2 |  |  | 28-May-02 | Georgia | Worth | USA | f | p | a | Adam Miles, Steven Castelberry |
| AM-15 | 2 |  |  | 28-May-02 | Georgia | Worth | USA | f | l | a | Adam Miles, Steven Castelberry |
| AM-55 | 2 |  |  | 05-Aug-02 | Georgia | Worth | USA | f | nr | a | Adam Miles, Steven Castelberry |
| IL-31 | 2 |  |  | 12-Aug-02 | Illinois | Clinton | USA | m |  | a | Tim Carter |
| IL-32 | 2 |  |  | 12-Aug-02 | Illinois | Clinton | USA | f |  | a | Tim Carter |
| IL-33 | 2 |  |  | 12-Aug-02 | Illinois | Clinton | USA | m |  | a | Tim Carter |
| IL-34 | 2 |  |  | 12-Aug-02 | Illinois | Clinton | USA | f |  | a | Tim Carter |
| IL-36 | 2 |  |  | 12-Aug-02 | Illinois | Clinton | USA | m |  | a | Tim Carter |
| IL-37 | 2 |  |  | 12-Aug-02 | Illinois | Clinton | USA | f |  | sa | Tim Carter |
| IL-38 | 2 |  |  | 12-Aug-02 | Illinois | Clinton | USA | f |  | sa | Tim Carter |
| IL-40 | 2 |  |  | 12-Aug-02 | Illinois | Clinton | USA | m |  | sa | Tim Carter |
| IL-23 | 2 |  |  | 08-Aug-02 | Illinois | Franklin | USA | m |  | a | Tim Carter |
| IL-28 | 2 |  |  | 08-Aug-02 | Illinois | Franklin | USA | m |  | sa | Tim Carter |
| IL-35 | 2 |  |  | 08-Aug-02 | Illinois | Franklin | USA | m |  | sa | Tim Carter |
| IL-41 | 2 |  |  | 08-Aug-02 | Illinois | Franklin | USA | m |  | a | Tim Carter |
| IL-01 | 2 |  |  | 28-May-02 | Illinois | Wayne | USA | m |  | a | Tim Carter |
| IL-02 | 2 |  |  | 30-May-02 | Illinois | Wayne | USA | m |  | a | Tim Carter |
| IL-05 | 2 |  |  | 30-May-02 | Illinois | Wayne | USA | f | p | a | Tim Carter |
| IL-07 | 2 |  |  | 31-May-02 | Illinois | Wayne | USA | f | p | a | Tim Carter |
| IL-10 | 2 |  |  | 10-Jul-02 | Illinois | Wayne | USA | f | l | a | Tim Carter |
| IL-11 | 2 |  |  | 12-Jul-02 | Illinois | Wayne | USA | f |  | j | Tim Carter |
| IL-16 | 2 |  |  | 13-Jul-02 | Illinois | Wayne | USA | m |  | j | Tim Carter |
| IL-17 | 2 |  |  | 05-Sep-02 | Illinois | Wayne | USA | m |  | a | Tim Carter |
| IL-18 | 2 |  |  | 13-Jul-02 | Illinois | Wayne | USA | f |  | j | Tim Carter |
| IL-19 | 2 |  |  | 13-Jul-02 | Illinois | Wayne | USA | f |  | j | Tim Carter |
| IL-20 | 2 |  |  | 13-Jul-02 | Illinois | Wayne | USA | f |  | a | Tim Carter |
| IL-29 | 2 |  |  | 07-Sep-02 | Illinois | Wayne | USA | m | s | a | Tim Carter |
| IL-42 | 2 |  |  | 08-Sep-02 | Illinois | Wayne | USA | m |  | a | Tim Carter |
| IL-43 | 2 |  |  | 05-Sep-02 | Illinois | Wayne | USA | m | s | a | Tim Carter |
| IL-44 | 2 |  |  | 06-Sep-02 | Illinois | Wayne | USA | m |  | a | Tim Carter |
| IL-46 | 2 |  |  | 06-Sep-02 | Illinois | Wayne | USA | m |  | a | Tim Carter |
| IL-47 | 2 |  |  | 08-Sep-02 | Illinois | Wayne | USA | m |  | a | Tim Carter |
| IL-48 | 2 |  |  | 06-Sep-02 | Illinois | Wayne | USA | m |  | a | Tim Carter |
| EB-197 | 2 |  |  | 08-Jun-02 | Kentucky | Ballard | USA | m | nr | a | Eric Britzke |
| JK-14 | 2 |  |  | 30-Jul-02 | Kentucky | Perry | USA | m | ns | a | James Kiser |
| JK-15 | 2 |  |  | 30-Jul-02 | Kentucky | Perry | USA | m | ns | a | James Kiser |
| EB-170 | 2 |  |  | 31-Jul-02 | Kentucky | Rowan | USA | m | s | a | Eric Britzke |
| EB-204 | 2 |  |  | 30-Jul-02 | Kentucky | Rowan | USA | m | s | a | Eric Britzke |
| EB-211 | 2 |  |  | 30-Jul-02 | Kentucky | Rowan | USA | m | s | a | Eric Britzke |
| EB-213 | 2 |  |  | 30-Jul-02 | Kentucky | Rowan | USA | f | pl | a | Eric Britzke |
| EB-215 | 2 |  |  | 31-Jul-02 | Kentucky | Rowan | USA | m | s | a | Eric Britzke |
| EB-44 | 2 |  |  | 29-May-02 | Kentucky | Rowan | USA | m | nr | a | Eric Britzke |
| EB-48 | 2 |  |  | 29-May-02 | Kentucky | Rowan | USA | m | nr | a | Eric Britzke |
| EB-63 | 2 |  |  | 04-Jun-02 | Kentucky | Rowan | USA | m | nr | a | Eric Britzke |
| EKPC-14 | 2 |  |  | 10-Aug-02 | Kentucky | Rowan | USA | m | s | j | Mark Gumbert |
| JK-18 | 2 |  |  | 05-Aug-02 | Kentucky | Trigg | USA | f | pl | a | James Kiser |
| JK-19 | 2 |  |  | 05-Aug-02 | Kentucky | Trigg | USA | f | pl | a | James Kiser |
| JK-20 | 2 |  |  | 05-Aug-02 | Kentucky | Trigg | USA | m | ns | a | James Kiser |
| JK-25 | 2 |  |  | 05-Aug-02 | Kentucky | Trigg | USA | f | pl | a | James Kiser |
| JK-26 | 2 |  |  | 05-Aug-02 | Kentucky | Trigg | USA | m |  | j | James Kiser |
| JK-27 | 2 |  |  | 05-Aug-02 | Kentucky | Trigg | USA | m |  | a | James Kiser |
| DB-08 | 2 |  |  | 11-Aug-02 | Kentucky |  | USA | m | s | a | Annie Tibbels |
| DB-09 | 2 |  |  | 12-Aug-02 | Kentucky |  | USA | m | s | a | Annie Tibbels |
| DB-10 | 2 |  |  | 12-Aug-02 | Kentucky |  | USA | m | s | j | Annie Tibbels |
| DB-11 | 2 |  |  | 12-Aug-02 | Kentucky |  | USA | m | s | j | Annie Tibbels |
| DB-12 | 2 |  |  | 12-Aug-02 | Kentucky |  | USA | m | s | a | Annie Tibbels |
| GJ-15 | 2 |  |  | 02-Aug-02 | Minnesota | Lincoln | USA | u |  | u | Greg Johnson |
| GJ-20 | 2 |  |  | 16-Aug-02 | Minnesota | Lincoln | USA | f |  | a | Greg Johnson |
| GJ-21 | 2 |  |  | 10-Sep-02 | Minnesota | Lincoln | USA | m |  | a | Greg Johnson |
| SA-164 | 2 |  |  | 10-Aug-02 | Missouri | Barry | USA | f | nr | a | Sybil Amelon |
| SA-167 | 2 |  |  | 12-Aug-02 | Missouri | Barry | USA | m | s | a | Sybil Amelon |
| SA-170 | 2 |  |  | 18-Aug-02 | Missouri | Carter | USA | m | s | a | Sybil Amelon |
| SA-171 | 2 |  |  | 18-Aug-02 | Missouri | Carter | USA | f | nr | a | Sybil Amelon |
| SA-172 | 2 |  |  | 18-Aug-02 | Missouri | Carter | USA | m | s | a | Sybil Amelon |
| SA-175 | 2 |  |  | 18-Aug-02 | Missouri | Carter | USA | f | nr | a | Sybil Amelon |
| SA-177 | 2 |  |  | 18-Aug-02 | Missouri | Carter | USA | m | s | a | Sybil Amelon |
| SA-180 | 2 |  |  | 18-Aug-02 | Missouri | Carter | USA | f | pl | a | Sybil Amelon |
| SA-225 | 2 |  |  | 26-Sep-02 | Missouri | Carter | USA | f | pl | a | Sybil Amelon |
| SA-226 | 2 |  |  | 26-Sep-02 | Missouri | Carter | USA | f | pl | a | Sybil Amelon |
| SA-227 | 2 |  |  | 26-Sep-02 | Missouri | Carter | USA | m | s | a | Sybil Amelon |
| SA-230 | 2 |  |  | 26-Sep-02 | Missouri | Carter | USA | m | s | a | Sybil Amelon |
| SA-231 | 2 |  |  | 26-Sep-02 | Missouri | Carter | USA | m | ss | a | Sybil Amelon |
| SA-233 | 2 |  |  | 26-Sep-02 | Missouri | Carter | USA | m | s | a | Sybil Amelon |
| SA-234 | 2 |  |  | 26-Sep-02 | Missouri | Carter | USA | m | s | a | Sybil Amelon |
| SA-236 | 2 |  |  | 26-Sep-02 | Missouri | Carter | USA | m | s | a | Sybil Amelon |
| SA-237 | 2 |  |  | 26-Sep-02 | Missouri | Carter | USA | m | s | a | Sybil Amelon |
| SA-238 | 2 |  |  | 26-Sep-02 | Missouri | Carter | USA | m | s | a | Sybil Amelon |
| SA-239 | 2 |  |  | 26-Sep-02 | Missouri | Carter | USA | m | s | a | Sybil Amelon |
| SA-240 | 2 |  |  | 26-Sep-02 | Missouri | Carter | USA | f | s | a | Sybil Amelon |
| SA-241 | 2 |  |  | 26-Sep-02 | Missouri | Carter | USA | m | s | a | Sybil Amelon |
| SA-243 | 2 |  |  | 26-Sep-02 | Missouri | Carter | USA | m | s | a | Sybil Amelon |
| SA-245 | 2 |  |  | 01-Oct-02 | Missouri | Carter | USA | m | s | a | Sybil Amelon |
| SA-246 | 2 |  |  | 01-Oct-02 | Missouri | Carter | USA | m | s | a | Sybil Amelon |
| SA-247 | 2 |  |  | 01-Oct-02 | Missouri | Carter | USA | m | s | a | Sybil Amelon |
| SA-249 | 2 |  |  | 01-Oct-02 | Missouri | Carter | USA | m | ss | a | Sybil Amelon |
| SA-250 | 2 |  |  | 01-Oct-02 | Missouri | Carter | USA | m | s | a | Sybil Amelon |
| SA-65 | 2 |  |  | 31-May-02 | Missouri | Howell | USA | m | ns | a | Sybil Amelon |
| SA-66 | 2 |  |  | 31-May-02 | Missouri | Howell | USA | m | ns | a | Sybil Amelon |
| SA-140 | 2 |  |  | 29-Jul-02 | Missouri | Phelps | USA | m | nr | a | Sybil Amelon |
| SA-152 | 2 |  |  | 04-Aug-02 | Missouri | Phelps | USA | f | nr | a | Sybil Amelon |
| SA-157 | 2 |  |  | 04-Aug-02 | Missouri | Phelps | USA | m | s | a | Sybil Amelon |
| SA-159 | 2 |  |  | 04-Aug-02 | Missouri | Phelps | USA | m | ns | a | Sybil Amelon |
| SA-160 | 2 |  |  | 04-Aug-02 | Missouri | Phelps | USA | m | s | a | Sybil Amelon |
| SA-169 | 2 |  |  | 14-Aug-02 | Missouri | Phelps | USA | f |  | a | Sybil Amelon |
| SA-207 | 2 |  |  | 17-Sep-02 | Missouri | Phelps | USA | m | s | a | Sybil Amelon |
| SA-208 | 2 |  |  | 17-Sep-02 | Missouri | Phelps | USA | m | s | a | Sybil Amelon |
| SA-210 | 2 |  |  | 17-Sep-02 | Missouri | Phelps | USA | m | s | a | Sybil Amelon |
| SA-211 | 2 |  |  | 17-Sep-02 | Missouri | Phelps | USA | m | s | a | Sybil Amelon |
| SA-212 | 2 |  |  | 17-Sep-02 | Missouri | Phelps | USA | m | s | a | Sybil Amelon |
| SA-213 | 2 |  |  | 17-Sep-02 | Missouri | Phelps | USA | m | s | a | Sybil Amelon |
| SA-215 | 2 |  |  | 17-Sep-02 | Missouri | Phelps | USA | m | s | a | Sybil Amelon |
| SA-217 | 2 |  |  | 17-Sep-02 | Missouri | Phelps | USA | m | s | a | Sybil Amelon |
| SA-218 | 2 |  |  | 17-Sep-02 | Missouri | Phelps | USA | m | s | a | Sybil Amelon |
| SA-220 | 2 |  |  | 17-Sep-02 | Missouri | Phelps | USA | m | s | a | Sybil Amelon |
| SA-221 | 2 |  |  | 17-Sep-02 | Missouri | Phelps | USA | m | s | a | Sybil Amelon |
| SA-222 | 2 |  |  | 17-Sep-02 | Missouri | Phelps | USA | m | s | a | Sybil Amelon |
| SA-223 | 2 |  |  | 17-Sep-02 | Missouri | Phelps | USA | m | s | a | Sybil Amelon |
| SA-252 | 2 |  |  | 22-Jul-02 | Missouri | Reynolds | USA | m |  | a | Sybil Amelon |
| SA-100 | 2 |  |  | 22-Jun-02 | Missouri | Texas | USA | f | l | a | Sybil Amelon |
| SA-181 | 2 |  |  | 21-Aug-02 | Missouri | Texas | USA | f | pl | a | Sybil Amelon |
| SA-182 | 2 |  |  | 21-Aug-02 | Missouri | Texas | USA | f | pl | a | Sybil Amelon |
| SA-195 | 2 |  |  | 30-Aug-02 | Missouri | Texas | USA | m | s | a | Sybil Amelon |
| SA-196 | 2 |  |  | 08-Sep-02 | Missouri | Texas | USA | f | nr | a | Sybil Amelon |
| SA-197 | 2 |  |  | 08-Sep-02 | Missouri | Texas | USA | f | nr | a | Sybil Amelon |
| SA-198 | 2 |  |  | 08-Sep-02 | Missouri | Texas | USA | f | nr | a | Sybil Amelon |
| SA-199 | 2 |  |  | 08-Sep-02 | Missouri | Texas | USA | m | s | a | Sybil Amelon |
| SA-200 | 2 |  |  | 08-Sep-02 | Missouri | Texas | USA | m | s | a | Sybil Amelon |
| SA-203 | 2 |  |  | 08-Sep-02 | Missouri | Texas | USA | m | s | a | Sybil Amelon |
| SA-204 | 2 |  |  | 08-Sep-02 | Missouri | Texas | USA | m | s | a | Sybil Amelon |
| SA-205 | 2 |  |  | 08-Sep-02 | Missouri | Texas | USA | m | s | a | Sybil Amelon |
| SA-251 | 2 |  |  | 09-Jul-02 | Missouri | Texas | USA | f | l | a | Sybil Amelon |
| FMSP-01 | 2 |  |  | 17-Jul-02 | North Carolina | Carteret | USA | m |  | j | Seth Lambiase |
| FMSP-02 | 2 |  |  | 17-Jul-02 | North Carolina | Carteret | USA | m |  | j | Seth Lambiase |
| TN-526 | 2 |  |  | 19-Mar-02 | North Carolina | Cocke | USA | m |  | a | Tennessee Rabies Lab |
| BMW-100 | 2 |  |  | 01-Oct-02 | Tennessee | Anderson | USA | m |  | a | Jenny Fiedler |
| BMW-101 | 2 |  |  | 01-Oct-02 | Tennessee | Anderson | USA | m |  | j | Jenny Fiedler |
| BMW-102 | 2 |  |  | 01-Oct-02 | Tennessee | Anderson | USA | m |  | a | Jenny Fiedler |
| BMW-103 | 2 |  |  | 01-Oct-02 | Tennessee | Anderson | USA | m |  | a | Jenny Fiedler |
| BMW-105 | 2 |  |  | 16-Sep-02 | Tennessee | Anderson | USA | m |  | j | Jenny Fiedler |
| BMW-106 | 2 |  |  | 16-Sep-02 | Tennessee | Anderson | USA | m |  | j | Jenny Fiedler |
| BMW-107 | 2 |  |  | 22-Sep-02 | Tennessee | Anderson | USA | m |  | j | Jenny Fiedler |
| BMW-108 | 2 |  |  | 04-Oct-02 | Tennessee | Anderson | USA | m |  | a | Jenny Fiedler |
| BMW-109 | 2 |  |  | 14-Oct-02 | Tennessee | Anderson | USA | m |  | a | Jenny Fiedler |
| BMW-110 | 2 |  |  | 14-Oct-02 | Tennessee | Anderson | USA | m |  | a | Jenny Fiedler |
| BMW-32 | 2 |  |  | 03-Jun-02 | Tennessee | Anderson | USA | m |  | j | Jenny Fiedler |
| BMW-34 | 2 |  |  | 06-Jun-02 | Tennessee | Anderson | USA | u |  |  | Jenny Fiedler |
| BMW-38 | 2 |  |  | 11-Jun-02 | Tennessee | Anderson | USA | m |  | j? | Jenny Fiedler |
| BMW-43 | 2 |  |  | 03-Jul-02 | Tennessee | Anderson | USA | u |  | u | Jenny Fiedler |
| BMW-47 | 2 |  |  | 26-Jul-02 | Tennessee | Anderson | USA | m |  | j? | Jenny Fiedler |
| BMW-49 | 2 |  |  | 31-Jul-02 | Tennessee | Anderson | USA | m |  | a | Jenny Fiedler |
| BMW-51 | 2 |  |  | 31-Jul-02 | Tennessee | Anderson | USA | m |  | j | Jenny Fiedler |
| BMW-52 | 2 |  |  | 31-Jul-02 | Tennessee | Anderson | USA | m |  | a | Jenny Fiedler |
| BMW-53 | 2 |  |  | 31-Jul-02 | Tennessee | Anderson | USA | m |  | j | Jenny Fiedler |
| BMW-57 | 2 |  |  | 31-Jul-02 | Tennessee | Anderson | USA | f |  | j | Jenny Fiedler |
| BMW-58 | 2 |  |  | 31-Jul-02 | Tennessee | Anderson | USA | m |  | j | Jenny Fiedler |
| BMW-59 | 2 |  |  | 31-Jul-02 | Tennessee | Anderson | USA | f |  | j | Jenny Fiedler |
| BMW-60 | 2 |  |  | 31-Jul-02 | Tennessee | Anderson | USA | m |  | j | Jenny Fiedler |
| BMW-61 | 2 |  |  | 31-Jul-02 | Tennessee | Anderson | USA | m |  | j | Jenny Fiedler |
| BMW-65 | 2 |  |  | 03-Sep-02 | Tennessee | Anderson | USA | m |  | a | Jenny Fiedler |
| BMW-66 | 2 |  |  | 03-Sep-02 | Tennessee | Anderson | USA | m |  | a | Jenny Fiedler |
| BMW-67 | 2 |  |  | 03-Sep-02 | Tennessee | Anderson | USA | m |  | a | Jenny Fiedler |
| BMW-68 | 2 |  |  | 03-Sep-02 | Tennessee | Anderson | USA | m |  | a | Jenny Fiedler |
| BMW-69 | 2 |  |  | 03-Sep-02 | Tennessee | Anderson | USA | m |  | j | Jenny Fiedler |
| BMW-70 | 2 |  |  | 03-Sep-02 | Tennessee | Anderson | USA | m |  | a | Jenny Fiedler |
| BMW-71 | 2 |  |  | 03-Sep-02 | Tennessee | Anderson | USA | m |  | a | Jenny Fiedler |
| BMW-73 | 2 |  |  | 11-Aug-02 | Tennessee | Anderson | USA | u |  | a | Jenny Fiedler |
| BMW-74 | 2 |  |  | 14-Aug-02 | Tennessee | Anderson | USA | m |  | j | Jenny Fiedler |
| BMW-76 | 2 |  |  | 23-Aug-02 | Tennessee | Anderson | USA | u |  | j | Jenny Fiedler |
| BMW-77 | 2 |  |  | 23-Aug-02 | Tennessee | Anderson | USA | f |  | j | Jenny Fiedler |
| BMW-78 | 2 |  |  | 23-Aug-02 | Tennessee | Anderson | USA | m |  | j | Jenny Fiedler |
| BMW-79 | 2 |  |  | 23-Aug-02 | Tennessee | Anderson | USA | u |  | j | Jenny Fiedler |
| BMW-80 | 2 |  |  | 23-Aug-02 | Tennessee | Anderson | USA | f |  | u | Jenny Fiedler |
| BMW-82 | 2 |  |  | 26-Aug-02 | Tennessee | Anderson | USA | f |  | a | Jenny Fiedler |
| BMW-83 | 2 |  |  | 26-Aug-02 | Tennessee | Anderson | USA | m |  | a | Jenny Fiedler |
| BMW-84 | 2 |  |  | 30-Aug-02 | Tennessee | Anderson | USA | m |  | a | Jenny Fiedler |
| BMW-86 | 2 |  |  | 30-Aug-02 | Tennessee | Anderson | USA | f |  | a | Jenny Fiedler |
| BMW-87 | 2 |  |  | 30-Aug-02 | Tennessee | Anderson | USA | m |  | a | Jenny Fiedler |
| BMW-91 | 2 |  |  | 31-Aug-02 | Tennessee | Anderson | USA | m |  | a | Jenny Fiedler |
| BMW-93 | 2 |  |  | 04-Sep-02 | Tennessee | Anderson | USA | m |  | a | Jenny Fiedler |
| BMW-94 | 2 |  |  | 04-Sep-02 | Tennessee | Anderson | USA | m |  | a | Jenny Fiedler |
| BMW-97 | 2 |  |  | 04-Sep-02 | Tennessee | Anderson | USA | m |  | a | Jenny Fiedler |
| BMW-98 | 2 |  |  | 04-Sep-02 | Tennessee | Anderson | USA | u |  | u | Jenny Fiedler |
| BMW-99 | 2 |  |  | 01-Oct-02 | Tennessee | Anderson | USA | m |  | a | Jenny Fiedler |
| TN-770 | 2 |  |  | 25-Jun-02 | Tennessee | Hamilton | USA | m |  | j | Tennessee Rabies Lab |
| TN-771 | 2 |  |  | 25-Jun-02 | Tennessee | Hamilton | USA | m |  | j | Tennessee Rabies Lab |
| TN-554 | 2 |  |  | 08-Apr-02 | Tennessee | Knox | USA | f |  |  | Tennessee Rabies Lab |
| TN-96 | 2 |  |  | 06-Aug-02 | Tennessee | Knox | USA | u |  |  | Tennessee Rabies Lab |
| TN-519 | 2 |  |  | 15-Mar-02 | Tennessee |  | USA | m |  | a | Tennessee Rabies Lab |
| TN-550 | 2 |  |  | 04-Apr-02 | Tennessee |  | USA | m |  |  | Tennessee Rabies Lab |
| TN-86 | 2 |  |  | 02-Aug-02 | Tennessee |  | USA | m |  |  | Tennessee Rabies Lab |
| CS28 | 2 |  |  | 16-Jul-02 | West Virginia | Mercer | USA | m | nr | a | Craig Stihler |
| CS29 | 2 |  |  | 16-Jul-02 | West Virginia | Mercer | USA | f | pl | a | Craig Stihler |
| CS39 | 2 |  |  | 17-Jul-02 | West Virginia | Mercer | USA | m | nr | a | Craig Stihler |
| CS41 | 2 |  |  | 17-Jul-02 | West Virginia | Mercer | USA | m | nr | a | Craig Stihler |
| CS78 | 2 |  |  | 24-Sep-02 | West Virginia | Pendleton | USA | m |  | j | Craig Stihler |
| CS79 | 2 |  |  | 24-Sep-02 | West Virginia | Pendleton | USA | m |  | j | Craig Stihler |
| EP-03 | 3 |  |  | 29-Jun-10 | Arkansas | Franklin | USA | m |  |  | Evan Pannkuk |
| EP-04 | 3 |  |  | 23-Jun-10 | Arkansas | Franklin | USA | m |  |  | Evan Pannkuk |
| EP-06 | 3 |  |  | 20-May-10 | Arkansas | Franklin | USA | f |  |  | Evan Pannkuk |
| EP-08 | 3 |  |  | 26-Jun-10 | Arkansas | Franklin | USA | m |  |  | Evan Pannkuk |
| EP-09 | 3 |  |  | 20-Jun-10 | Arkansas | Franklin | USA |  |  |  | Evan Pannkuk |
| EP-10 | 3 |  |  | 30-Jun-10 | Arkansas | Franklin | USA |  |  |  | Evan Pannkuk |
| EP-13 | 3 |  |  | 30-Jun-10 | Arkansas | Franklin | USA |  |  |  | Evan Pannkuk |
| EP-17 | 3 |  |  | 30-Jun-10 | Arkansas | Franklin | USA |  |  |  | Evan Pannkuk |
| EP-18 | 3 |  |  | 30-Jun-10 | Arkansas | Franklin | USA |  |  |  | Evan Pannkuk |
| EP-19 | 3 |  |  | 30-Jun-10 | Arkansas | Franklin | USA |  |  |  | Evan Pannkuk |
| EP-20 | 3 |  |  | 30-Jun-10 | Arkansas | Franklin | USA |  |  |  | Evan Pannkuk |
| EP-21 | 3 |  |  | 30-Jun-10 | Arkansas | Franklin | USA |  |  |  | Evan Pannkuk |
| EP-22 | 3 |  |  | 30-Jun-10 | Arkansas | Franklin | USA |  |  |  | Evan Pannkuk |
| EP-23 | 3 |  |  | 23-Jun-10 | Arkansas | Franklin | USA |  |  |  | Evan Pannkuk |
| EP-24 | 3 |  |  | 20-Jun-10 | Arkansas | Franklin | USA |  |  |  | Evan Pannkuk |
| EP-27 | 3 |  |  | 22-Jun-10 | Arkansas | Franklin | USA | m |  |  | Evan Pannkuk |
| EP-28 | 3 |  |  | 20-Jun-10 | Arkansas | Franklin | USA |  |  |  | Evan Pannkuk |
| EP-29 | 3 |  |  | 20-Jun-10 | Arkansas | Franklin | USA |  |  |  | Evan Pannkuk |
| EP-31 | 3 |  |  | 30-Jun-10 | Arkansas | Franklin | USA |  |  |  | Evan Pannkuk |
| EP-34 | 3 |  |  | 30-Jun-10 | Arkansas | Franklin | USA |  |  |  | Evan Pannkuk |
| EP-35 | 3 |  |  | 20-Jun-10 | Arkansas | Franklin | USA |  |  |  | Evan Pannkuk |
| EP-38 | 3 |  |  | 20-Jun-10 | Arkansas | Franklin | USA |  |  |  | Evan Pannkuk |
| EP-50 | 3 |  |  | 30-Jun-10 | Arkansas | Franklin | USA |  |  |  | Evan Pannkuk |
| AR-135 | 3 |  |  | 12-Aug-10 | Arkansas | Garland | USA | m | s | a | David Saugey |
| AR-136 | 3 |  |  | 12-Aug-10 | Arkansas | Garland | USA | m | s | a | David Saugey |
| AR-137 | 3 |  |  | 12-Aug-10 | Arkansas | Garland | USA | m | s | a | David Saugey |
| AR-138 | 3 |  |  | 12-Aug-10 | Arkansas | Garland | USA | m | s | a | David Saugey |
| AR-139 | 3 |  |  | 12-Aug-10 | Arkansas | Garland | USA | m | s | a | David Saugey |
| AR-140 | 3 |  |  | 21-Sep-10 | Arkansas | Garland | USA | m | s | a | David Saugey |
| AR-141 | 3 |  |  | 21-Sep-10 | Arkansas | Garland | USA | m | s | a | David Saugey |
| AR-142 | 3 |  |  | 21-Sep-10 | Arkansas | Garland | USA | m | s | a | David Saugey |
| AR-143 | 3 |  |  | 21-Sep-10 | Arkansas | Garland | USA | m | s | a | David Saugey |
| AR-144 | 3 |  |  | 21-Sep-10 | Arkansas | Garland | USA | f |  | a | David Saugey |
| AR-145 | 3 |  |  | 21-Sep-10 | Arkansas | Garland | USA | m | s | a | David Saugey |
| AR-146 | 3 |  |  | 21-Sep-10 | Arkansas | Garland | USA | m | s | a | David Saugey |
| AR-147 | 3 |  |  | 21-Sep-10 | Arkansas | Garland | USA | m | s | a | David Saugey |
| AR-148 | 3 |  |  | 21-Sep-10 | Arkansas | Garland | USA | f |  | a | David Saugey |
| AR-149 | 3 |  |  | 21-Sep-10 | Arkansas | Garland | USA | f |  | a | David Saugey |
| AR-160 | 3 |  |  | 30-Sep-10 | Arkansas | Garland | USA | m | s | a | David Saugey |
| AR-161 | 3 |  |  | 30-Sep-10 | Arkansas | Garland | USA | m | s | a | David Saugey |
| AR-162 | 3 |  |  | 30-Sep-10 | Arkansas | Garland | USA | m | s | a | David Saugey |
| AR-163 | 3 |  |  | 30-Sep-10 | Arkansas | Garland | USA | m | s | a | David Saugey |
| AR-164 | 3 |  |  | 30-Sep-10 | Arkansas | Garland | USA | m | s | a | David Saugey |
| AR-165 | 3 |  |  | 30-Sep-10 | Arkansas | Garland | USA | f |  | a | David Saugey |
| AR-167 | 3 |  |  | 30-Sep-10 | Arkansas | Garland | USA | f |  | a | David Saugey |
| AR-168 | 3 |  |  | 30-Sep-10 | Arkansas | Garland | USA | f |  | a | David Saugey |
| AR-169 | 3 |  |  | 30-Sep-10 | Arkansas | Garland | USA | m | s | a | David Saugey |
| AR-170 | 3 |  |  | 30-Sep-10 | Arkansas | Garland | USA | m | s | a | David Saugey |
| EP-01 | 3 |  |  | 20-Jun-10 | Arkansas | Johnson | USA | m |  |  | Evan Pannkuk |
| EP-02 | 3 |  |  | 04-Aug-10 | Arkansas | Johnson | USA | f |  |  | Evan Pannkuk |
| EP-05 | 3 |  |  | 04-Aug-10 | Arkansas | Johnson | USA | m |  |  | Evan Pannkuk |
| EP-07 | 3 |  |  | 01-Aug-10 | Arkansas | Johnson | USA | F |  |  | Evan Pannkuk |
| EP-11 | 3 |  |  | 06-Aug-10 | Arkansas | Johnson | USA | m |  |  | Evan Pannkuk |
| EP-12 | 3 |  |  | 06-Aug-10 | Arkansas | Johnson | USA | m |  |  | Evan Pannkuk |
| EP-14 | 3 |  |  | 06-Aug-10 | Arkansas | Johnson | USA | f |  |  | Evan Pannkuk |
| EP-15 | 3 |  |  | 06-Aug-10 | Arkansas | Johnson | USA | f |  |  | Evan Pannkuk |
| EP-16 | 3 |  |  | 06-Aug-10 | Arkansas | Johnson | USA | f |  |  | Evan Pannkuk |
| EP-25 | 3 |  |  | 04-Aug-10 | Arkansas | Johnson | USA | f |  |  | Evan Pannkuk |
| EP-26 | 3 |  |  | 04-Aug-10 | Arkansas | Johnson | USA | f |  |  | Evan Pannkuk |
| EP-30 | 3 |  |  | 03-Aug-10 | Arkansas | Johnson | USA | f |  |  | Evan Pannkuk |
| EP-32 | 3 |  |  | 04-Aug-10 | Arkansas | Johnson | USA | m |  |  | Evan Pannkuk |
| EP-33 | 3 |  |  | 01-Aug-10 | Arkansas | Johnson | USA |  |  |  | Evan Pannkuk |
| EP-36 | 3 |  |  | 04-Aug-10 | Arkansas | Johnson | USA | m |  |  | Evan Pannkuk |
| EP-37 | 3 |  |  | 04-Aug-10 | Arkansas | Johnson | USA | m |  |  | Evan Pannkuk |
| EP-39 | 3 |  |  | 04-Aug-10 | Arkansas | Johnson | USA | m |  |  | Evan Pannkuk |
| EP-40 | 3 |  |  | 30-Sep-10 | Arkansas | Johnson | USA | f |  |  | Evan Pannkuk |
| EP-41 | 3 |  |  | 30-Jul-10 | Arkansas | Johnson | USA | f |  |  | Evan Pannkuk |
| EP-42 | 3 |  |  | 03-Aug-10 | Arkansas | Johnson | USA | f |  |  | Evan Pannkuk |
| EP-43 | 3 |  |  | 03-Aug-10 | Arkansas | Johnson | USA | f |  |  | Evan Pannkuk |
| EP-44 | 3 |  |  | 03-Aug-10 | Arkansas | Johnson | USA | f |  |  | Evan Pannkuk |
| EP-45 | 3 |  |  | 03-Aug-10 | Arkansas | Johnson | USA | f |  |  | Evan Pannkuk |
| EP-46 | 3 |  |  | 04-Aug-10 | Arkansas | Johnson | USA | m |  |  | Evan Pannkuk |
| EP-47 | 3 |  |  | 31-Jul-10 | Arkansas | Johnson | USA | f |  |  | Evan Pannkuk |
| EP-48 | 3 |  |  | 04-Aug-10 | Arkansas | Johnson | USA | m |  |  | Evan Pannkuk |
| EP-49 | 3 |  |  | 04-Aug-10 | Arkansas | Johnson | USA | m |  |  | Evan Pannkuk |
| AR-150 | 3 |  |  | 23-Sep-10 | Arkansas | Perry | USA | m | s | a | David Saugey |
| AR-151 | 3 |  |  | 23-Sep-10 | Arkansas | Perry | USA | m | s | a | David Saugey |
| AR-152 | 3 |  |  | 23-Sep-10 | Arkansas | Perry | USA | m | s | a | David Saugey |
| AR-153 | 3 |  |  | 23-Sep-10 | Arkansas | Perry | USA | m | s | a | David Saugey |
| AR-154 | 3 |  |  | 23-Sep-10 | Arkansas | Perry | USA | m | s | a | David Saugey |
| AR-155 | 3 |  |  | 23-Sep-10 | Arkansas | Perry | USA | m | s | a | David Saugey |
| AR-156 | 3 |  |  | 23-Sep-10 | Arkansas | Perry | USA | m | s | a | David Saugey |
| AR-157 | 3 |  |  | 23-Sep-10 | Arkansas | Perry | USA | m | s | a | David Saugey |
| AR-158 | 3 |  |  | 23-Sep-10 | Arkansas | Perry | USA | m | s | a | David Saugey |
| AR-159 | 3 |  |  | 23-Sep-10 | Arkansas | Perry | USA | f |  | a | David Saugey |
| MG-196 | 3 |  |  | 10-Jul-10 | Illinois | Ford | USA | f | nr | j | Mark Gumbert |
| MG-197 | 3 |  |  | 10-Jul-10 | Illinois | Ford | USA | f | nr | j | Mark Gumbert |
| MG-198 | 3 |  |  | 10-Jul-10 | Illinois | Ford | USA | f | nr | j | Mark Gumbert |
| MG-199 | 3 |  |  | 11-Jul-10 | Illinois | Ford | USA | m | s | a | Mark Gumbert |
| MG-200 | 3 |  |  | 11-Jul-10 | Illinois | Ford | USA | m | s | a | Mark Gumbert |
| MG-201 | 3 |  |  | 11-Jul-10 | Illinois | Ford | USA | f | nr | j | Mark Gumbert |
| MG-202 | 3 |  |  | 11-Jul-10 | Illinois | Ford | USA | f | nr | j | Mark Gumbert |
| MG-194 | 3 |  |  | 08-Jul-10 | Illinois | Vermilion | USA | f | pl | a | Mark Gumbert |
| MG-195 | 3 |  |  | 09-Jul-10 | Illinois | Vermilion | USA | m | s | a | Mark Gumbert |
| MG-203 | 3 |  |  | 27-Jul-10 | Indiana | Bartholomew | USA | m | nr | a | Mark Gumbert |
| MG-204 | 3 |  |  | 27-Jul-10 | Indiana | Bartholomew | USA | m | nr | j | Mark Gumbert |
| MG-205 | 3 |  |  | 28-Jul-10 | Indiana | Bartholomew | USA | f | nr | j | Mark Gumbert |
| MG-206 | 3 |  |  | 28-Jul-10 | Indiana | Bartholomew | USA | m | nr | j | Mark Gumbert |
| MG-207 | 3 |  |  | 29-Jul-10 | Indiana | Bartholomew | USA | m | s | j | Mark Gumbert |
| MG-208 | 3 |  |  | 31-Jul-10 | Indiana | Bartholomew | USA | f | nr | j | Mark Gumbert |
| MG-209 | 3 |  |  | 31-Jul-10 | Indiana | Bartholomew | USA | m | s | a | Mark Gumbert |
| MG-210 | 3 |  |  | 04-Aug-10 | Indiana | Bartholomew | USA | m | nr | j | Mark Gumbert |
| MG-211 | 3 |  |  | 04-Aug-10 | Indiana | Bartholomew | USA | m | nr | j | Mark Gumbert |
| MG-212 | 3 |  |  | 04-Aug-10 | Indiana | Bartholomew | USA | m | s | j | Mark Gumbert |
| MG-213 | 3 |  |  | 04-Aug-10 | Indiana | Bartholomew | USA | m | nr | j | Mark Gumbert |
| LP-01 | 3 |  |  | 16-Aug-10 | Indiana | Benton | USA | m |  | u | Lori Pruitt |
| LP-02 | 3 |  |  | 16-Aug-10 | Indiana | Benton | USA | m |  | u | Lori Pruitt |
| LP-03 | 3 |  |  | 17-Aug-10 | Indiana | Benton | USA | f |  | a | Lori Pruitt |
| LP-04 | 3 |  |  | 17-Aug-10 | Indiana | Benton | USA | u |  | u | Lori Pruitt |
| LP-05 | 3 |  |  | 17-Aug-10 | Indiana | Benton | USA | u |  | u | Lori Pruitt |
| MG-189 | 3 |  |  | 12-Jul-10 | Indiana | Montgomery | USA | m | s | a | Mark Gumbert |
| MG-190 | 3 |  |  | 12-Jul-10 | Indiana | Montgomery | USA | f | nr | j | Mark Gumbert |
| MG-191 | 3 |  |  | 12-Jul-10 | Indiana | Montgomery | USA | f | pl | a | Mark Gumbert |
| MG-192 | 3 |  |  | 14-Jul-10 | Indiana | Montgomery | USA | f | nr | j | Mark Gumbert |
| MG-187 | 3 |  |  | 03-Jul-10 | Kentucky | Bullitt | USA | f | l | a | Mark Gumbert |
| MG-188 | 3 |  |  | 03-Jul-10 | Kentucky | Bullitt | USA | m |  | a | Mark Gumbert |
| LR-35 | 3 |  |  | 01-Aug-10 | Missouri | Carter | USA | m | s | a | Lynn Robbins |
| LR-36 | 3 |  |  | 01-Aug-10 | Missouri | Carter | USA | f | nr | a | Lynn Robbins |
| LR-37 | 3 |  |  | 01-Aug-10 | Missouri | Carter | USA | m | s | a | Lynn Robbins |
| LR-38 | 3 |  |  | 01-Aug-10 | Missouri | Carter | USA | m | nr | a | Lynn Robbins |
| LR-39 | 3 |  |  | 01-Aug-10 | Missouri | Carter | USA | m | nr | a | Lynn Robbins |
| LR-40 | 3 |  |  | 01-Aug-10 | Missouri | Carter | USA | m | s | a | Lynn Robbins |
| LR-41 | 3 |  |  | 01-Aug-10 | Missouri | Carter | USA | f | nr | j | Lynn Robbins |
| LR-42 | 3 |  |  | 01-Aug-10 | Missouri | Carter | USA | m | nr | a | Lynn Robbins |
| LR-10 | 3 |  |  | 06-Jul-10 | Missouri | Franklin | USA | f | nr | j | Lynn Robbins |
| LR-06 | 3 |  |  | 24-Jun-10 | Missouri | Macon | USA | f | l | a | Lynn Robbins |
| LR-07 | 3 |  |  | 24-Jun-10 | Missouri | Macon | USA | f | l | a | Lynn Robbins |
| LR-15 | 3 |  |  | 15-Jul-10 | Missouri | Schulyer | USA | f | nr | j | Lynn Robbins |
| LR-17 | 3 |  |  | 17-Jul-10 | Missouri | Schulyer | USA | f | nr | j | Lynn Robbins |
| LR-18 | 3 |  |  | 15-Jul-10 | Missouri | Schulyer | USA | f | nr | j | Lynn Robbins |
| LR-19 | 3 |  |  | 17-Jul-10 | Missouri | Schulyer | USA | m | nr | j | Lynn Robbins |
| LR-22 | 3 |  |  | 15-Jul-10 | Missouri | Schulyer | USA | m | nr | j | Lynn Robbins |
| LR-23 | 3 |  |  | 15-Jul-10 | Missouri | Schulyer | USA | f | nr | j | Lynn Robbins |
| LR-24 | 3 |  |  | 15-Jul-10 | Missouri | Schulyer | USA | f | nr | j | Lynn Robbins |
| LR-26 | 3 |  |  | 15-Jul-10 | Missouri | Schulyer | USA | f | nr | j | Lynn Robbins |
| LR-27 | 3 |  |  | 25-Jul-10 | Missouri | Shannon | USA | m | s | a | Lynn Robbins |
| LR-28 | 3 |  |  | 25-Jul-10 | Missouri | Shannon | USA | f | nr | a | Lynn Robbins |
| LR-29 | 3 |  |  | 25-Jul-10 | Missouri | Shannon | USA | m | ns | a | Lynn Robbins |
| LR-30 | 3 |  |  | 23-Jul-10 | Missouri | Shannon | USA | f | nr | j | Lynn Robbins |
| LR-33 | 3 |  |  | 05-Sep-10 | Missouri | Shannon | USA | m | s | a | Lynn Robbins |
| LR-43 | 3 |  |  | 30-Jul-10 | Missouri | Shannon | USA | m | s | a | Lynn Robbins |
| LR-44 | 3 |  |  | 30-Jul-10 | Missouri | Shannon | USA | f | nr | a | Lynn Robbins |
| LR-45 | 3 |  |  | 30-Jul-10 | Missouri | Shannon | USA | m | s | a | Lynn Robbins |
| LR-46 | 3 |  |  | 30-Jul-10 | Missouri | Shannon | USA | f | nr | a | Lynn Robbins |
| LR-47 | 3 |  |  | 30-Jul-10 | Missouri | Shannon | USA | m | s | a | Lynn Robbins |
| LR-48 | 3 |  |  | 04-Sep-10 | Missouri | Shannon | USA | m | s | a | Lynn Robbins |
| LR-49 | 3 |  |  | 23-Oct-10 | Missouri | Shannon | USA | m | s | a | Lynn Robbins |
| LR-50 | 3 |  |  | 31-Jul-10 | Missouri | Shannon | USA | m | s | a | Lynn Robbins |
| LR-51 | 3 |  |  | 31-Jul-10 | Missouri | Shannon | USA | f | nr | a | Lynn Robbins |
| LR-52 | 3 |  |  | 31-Jul-10 | Missouri | Shannon | USA | m | s | a | Lynn Robbins |
| LR-53 | 3 |  |  | 31-Jul-10 | Missouri | Shannon | USA | m | nr | a | Lynn Robbins |
| LR-54 | 3 |  |  | 31-Jul-10 | Missouri | Shannon | USA | m | nr | a | Lynn Robbins |
| LR-55 | 3 |  |  | 31-Jul-10 | Missouri | Shannon | USA | m | s | a | Lynn Robbins |
| LR-56 | 3 |  |  | 31-Jul-10 | Missouri | Shannon | USA | m | s | a | Lynn Robbins |
| LR-57 | 3 |  |  | 31-Jul-10 | Missouri | Shannon | USA | m | s | a | Lynn Robbins |
| LR-58 | 3 |  |  | 31-Jul-10 | Missouri | Shannon | USA | f | nr | a | Lynn Robbins |
| LR-59 | 3 |  |  | 31-Jul-10 | Missouri | Shannon | USA | m | nr | a | Lynn Robbins |
| LR-60 | 3 |  |  | 23-Oct-10 | Missouri | Shannon | USA | m | nr | a | Lynn Robbins |
| LR-61 | 3 |  |  | 23-Oct-10 | Missouri | Shannon | USA | m | nr | a | Lynn Robbins |
| LR-63 | 3 |  |  | 28-Aug-10 | Missouri | Shannon | USA | f | nr | a | Lynn Robbins |
| LR-64 | 3 |  |  | 28-Aug-10 | Missouri | Shannon | USA | m | s | a | Lynn Robbins |
| LR-65 | 3 |  |  | 28-Aug-10 | Missouri | Shannon | USA | m | s | a | Lynn Robbins |
| LR-66 | 3 |  |  | 28-Aug-10 | Missouri | Shannon | USA | m | s | a | Lynn Robbins |
| LR-67 | 3 |  |  | 28-Aug-10 | Missouri | Shannon | USA | m | s | a | Lynn Robbins |
| LR-68 | 3 |  |  | 24-Oct-10 | Missouri | Shannon | USA | m | s | a | Lynn Robbins |
| LR-70 | 3 |  |  | 01-Oct-10 | Missouri | Shannon | USA | m | s | a | Lynn Robbins |
| LR-71 | 3 |  |  | 01-Oct-10 | Missouri | Shannon | USA | m | s | a | Lynn Robbins |
| LR-72 | 3 |  |  | 01-Oct-10 | Missouri | Shannon | USA | f | nr | a | Lynn Robbins |
| LR-73 | 3 |  |  | 10-Sep-10 | Missouri | Shannon | USA | m | s | a | Lynn Robbins |
| LR-74 | 3 |  |  | 06-Aug-10 | Missouri | Shannon | USA | m | s | a | Lynn Robbins |
| LR-75 | 3 |  |  | 06-Aug-10 | Missouri | Shannon | USA | f | nr | a | Lynn Robbins |
| LR-76 | 3 |  |  | 11-Sep-10 | Missouri | Shannon | USA | m | s | a | Lynn Robbins |
| LR-77 | 3 |  |  | 11-Sep-10 | Missouri | Shannon | USA | m | s | a | Lynn Robbins |
| LR-78 | 3 |  |  | 11-Sep-10 | Missouri | Shannon | USA | m | s | a | Lynn Robbins |
| LR-79 | 3 |  |  | 11-Sep-10 | Missouri | Shannon | USA | m | s | a | Lynn Robbins |
| LR-83 | 3 |  |  | 10-Nov-10 | Missouri | Shannon | USA | m | s | a | Lynn Robbins |
| LR-84 | 3 |  |  | 10-Nov-10 | Missouri | Shannon | USA | m | s | a | Lynn Robbins |
| LR-87 | 3 |  |  | 24-Sep-10 | Missouri | Shannon | USA | m | s | a | Lynn Robbins |
| DT-48 | 3 |  |  | 07-Jul-10 | North Carolina | Buncombe | USA | m | nr | a | Dottie Brown |
| DT-49 | 3 |  |  | 07-Jul-10 | North Carolina | Buncombe | USA | f | nr | j | Dottie Brown |
| DT-50 | 3 |  |  | 07-Jul-10 | North Carolina | Buncombe | USA | f | l | a | Dottie Brown |
| DT-05 | 3 |  |  | 21-Jun-10 | North Carolina | Graham | USA | m | nr | a | Dottie Brown |
| DT-07 | 3 |  |  | 21-Jun-10 | North Carolina | Graham | USA | m | nr | a | Dottie Brown |
| DT-08 | 3 |  |  | 21-Jun-10 | North Carolina | Graham | USA | m | nr | a | Dottie Brown |
| DT-30 | 3 |  |  | 01-Jul-10 | North Carolina | Haywood | USA | m | nr | a | Dottie Brown |
| DT-31 | 3 |  |  | 01-Jul-10 | North Carolina | Haywood | USA | m | nr | a | Dottie Brown |
| JO-276 | 3 |  |  | 26-May-10 | North Carolina | Cherokee | USA | m | nr | a | Joy O'Keefe |
| JO-278 | 3 |  |  | 27-May-10 | North Carolina | Cherokee | USA | m | nr | a | Joy O'Keefe |
| JO-286 | 3 |  |  | 18-Jun-10 | North Carolina | Graham | USA | m | nr | a | Joy O'Keefe |
| JO-287 | 3 |  |  | 18-Jun-10 | North Carolina | Graham | USA | m | nr | a | Joy O'Keefe |
| JO-293 | 3 |  |  | 30-Jun-10 | North Carolina | Swain | USA | f | pl | a | Joy O'Keefe |
| MG-170 | 3 |  |  | 14-Jul-10 | Penssylvania | Lycoming | USA | m |  | a | Mark Gumbert |
| MG-171 | 3 |  |  | 15-Jul-10 | Penssylvania | Lycoming | USA | m |  | a | Mark Gumbert |
| MG-172 | 3 |  |  | 26-Jul-10 | Penssylvania | Lycoming | USA | m |  | a | Mark Gumbert |
| MG-173 | 3 |  |  | 23-Jul-10 | Penssylvania | Lycoming | USA | m |  | a | Mark Gumbert |
| MG-174 | 3 |  |  | 28-Jul-10 | Penssylvania | Lycoming | USA | m |  | a | Mark Gumbert |
| MG-175 | 3 |  |  | 30-Jul-10 | Penssylvania | Lycoming | USA | m | s | a | Mark Gumbert |
| MG-176 | 3 |  |  | 02-Aug-10 | Penssylvania | Lycoming | USA | m | s | a | Mark Gumbert |
| MG-177 | 3 |  |  | 02-Aug-10 | Penssylvania | Lycoming | USA | m | s | j | Mark Gumbert |
| MG-178 | 3 |  |  | 03-Aug-10 | Penssylvania | Lycoming | USA | f |  | j | Mark Gumbert |
| MG-179 | 3 |  |  | 03-Aug-10 | Penssylvania | Lycoming | USA | m | nr | a | Mark Gumbert |
| MG-180 | 3 |  |  | 08-Aug-10 | Penssylvania | Lycoming | USA | m | nr | a | Mark Gumbert |
| MG-181 | 3 |  |  | 08-Aug-10 | Penssylvania | Lycoming | USA | m | s | j | Mark Gumbert |
| MG-182 | 3 |  |  | 11-Aug-10 | Penssylvania | Lycoming | USA | m | s | a | Mark Gumbert |
| MG-183 | 3 |  |  | 13-Aug-10 | Penssylvania | Lycoming | USA | m | nr | j | Mark Gumbert |
| MG-184 | 3 |  |  | 13-Aug-10 | Penssylvania | Lycoming | USA | m | nr | j | Mark Gumbert |
| MG-185 | 3 |  |  | 13-Aug-10 | Penssylvania | Lycoming | USA | f | nr | j | Mark Gumbert |
| MG-186 | 3 |  |  | 14-Aug-10 | Penssylvania | Lycoming | USA | f | nr | j | Mark Gumbert |
| PA-177 | 3 |  |  | 07-Aug-10 | Penssylvania | Somerset | USA | m |  | a | Michael Schirmacher |
| PA-181 | 3 |  |  | 08-Aug-10 | Penssylvania |  | USA | m |  | a | Michael Schirmacher |
| JO-294 | 3 |  |  | 07-Jul-10 | Tennessee | Blount | USA | m | nr | j | Joy O'Keefe |
| JO-300 | 3 |  |  | 11-Jul-10 | Tennessee | Blount | USA | m | s | a | Joy O'Keefe |
| JO-302 | 3 |  |  | 14-Jul-10 | Tennessee | Blount | USA | m | nr | a | Joy O'Keefe |
| JO-304 | 3 |  |  | 14-Jul-10 | Tennessee | Blount | USA | m | nr | a | Joy O'Keefe |
| JO-310 | 3 |  |  | 29-Jul-10 | Tennessee | Blount | USA | m | nr | a | Joy O'Keefe |
| JO-318 | 3 |  |  | 06-Aug-10 | Tennessee | Blount | USA | f | nr | j | Joy O'Keefe |
| JO-320 | 3 |  |  | 06-Aug-10 | Tennessee | Blount | USA | m | s | a | Joy O'Keefe |
| JO-321 | 3 |  |  | 06-Aug-10 | Tennessee | Blount | USA | m | s | a | Joy O'Keefe |
| JO-322 | 3 |  |  | 06-Aug-10 | Tennessee | Blount | USA | f | nr | j | Joy O'Keefe |
| MG-193 | 3 |  |  | 06-Jul-10 | Tennessee | Claibourne | USA | f | pl | a | Mark Gumbert |
| WT-01 | 3 |  |  | 23-Jul-10 |  |  | USA |  |  |  | Wendy Tidhar |
| WT-02 | 3 |  |  | 07-Jul-10 |  |  | USA |  |  |  | Wendy Tidhar |
| WT-04 | 3 |  |  | 30-Jul-10 |  |  | USA | f |  |  | Wendy Tidhar |
| WT-06 | 3 |  |  | 28-Aug-10 |  |  | USA |  |  |  | Wendy Tidhar |
| WT-07 | 3 |  |  | 23-Jul-10 |  |  | USA |  |  |  | Wendy Tidhar |
