# Supplementary material for: Genetic approaches to the conservation of migratory bats: a study of the eastern red bat (Lasiurus borealis)
Source: PeerJ. 2015 May 28;3:e983. doi: 10.7717/peerj.983 (PMC4451038; doi:10.7717/peerj.983)
Supplement: Table S2 — Listed are load (combined PCR amplifications loaded on an ABI 3130 sequencer), PCR (multiplex panel and single-locus amplifications), annealing temperature (Ta), whether or not an extension is required (‘yes’ requires a final 30 min extension at 72 °C, ‘no’ skips this step), the number of cycles (we used a two-step PCR: the basic conditions started with 3 cycles, followed by 33 or 39 cycles [see main text]), locus, fluorophore, volume of each primer in PCR reaction (using GE Healthcare Illustra Pure-Taq Ready-to-Go Beads, we added 2 µl template, the listed volume of each primer, and water to a total volume of 25 µl), and the citation for the locus. [file peerj-03-983-s002.docx]

Table S2. Locus information for 16 microsatellites used to genotype *L. borealis*. Listed are load (combined PCR amplifications loaded on an ABI 3130 sequencer), PCR (multiplex panel and single-locus amplifications), annealing temperature (T_a_), whether or not an extension is required (‘yes’ requires a final 30 min extension at 72 °C, ‘no’ skips this step), the number of cycles (we used a two-step PCR: the basic conditions started with 3 cycles, followed by 33 or 39 cycles [see main text]), locus, fluorophore, volume of each primer in PCR reaction (using GE Healthcare Illustra Pure-Taq Ready-to-Go Beads, we added 2 μl template, the listed volume of each primer, and water to a total volume of 25 μl), and the citation for the locus.

| Load | PCR | T_a_ (°C) | Extension | PCR Cycles | Locus | Fluorophore | Volume (μl) | Citation |
| --- | --- | --- | --- | --- | --- | --- | --- | --- |
| 1 | 1 | 54 | yes | 33 | LboD226 | 6-FAM | 0.5 | Eackles & King, Pers. Comm. |
|  |  |  |  |  | LboD200 | VIC | 0.6 | Eackles & King, Pers. Comm. |
|  | 2 | 54 | yes | 33 | LboD202 | PET | 0.5 | Eackles & King, Pers. Comm. |
|  |  |  |  |  | LboC07 | NED | 0.4 | Eackles & King, Pers. Comm. |
|  | 3 | 60 | no | 33 | Coto_G12F_B11R | 6-FAM | 1 | Piaggio et al., 2009b |
| 2 | 4 | 54 | yes | 33 | LboD203 | 6-FAM | 0.8 | Eackles & King, Pers. Comm. |
|  |  |  |  |  | LboD204 | NED | 0.8 | Eackles & King, Pers. Comm. |
|  |  |  |  |  | LboD248 | VIC | 1 | Eackles & King, Pers. Comm. |
|  | 5 | 54 | no | 39 | IBat CA22 | PET | 1 | Oyler-McCance et al., 2011 |
|  | 6 | 54 | yes | 33 | Cora_F11_C04 | VIC | 1 | Piaggio, Figueroa & Perkins, 2009a |
| 3 | 7 | 54 | yes | 33 | LboB06 | 6-FAM | 1 | Eackles & King, Pers. Comm. |
|  |  |  |  |  | LboD240 | PET | 0.8 | Eackles & King, Pers. Comm. |
|  | 8 | 54 | yes | 33 | LboD245 | NED | 0.5 | Eackles & King, Pers. Comm. |
|  |  |  |  |  | LboD08 | VIC | 1 | Eackles & King, Pers. Comm. |
|  | 9 | 60 | yes | 33 | MS1C01 | NED | 1 | Trujillo & Amelon, 2009 |
|  | 10 | 60 | yes | 39 | MS3E10 | 6-FAM | 1 | Trujillo & Amelon 2009 |

**Piaggio AJ, Figueroa JA, Perkins SL. 2009a.** Development and Characterization of 15 Polymorphic Microsatellite Loci Isolated from Rafinesque's Big-Eared Bat, *Corynorhinus rafinesquii*. *Molecular Ecology Resources* **9:**1191-1193 DOI 10.1111/j.1755-0998-2009-02625.x.

**Piaggio AJ, Miller KEG, Matocq MD, Perkins SL. 2009b.** Eight Polymorphic Microsatellite Loci Developed and Characterized from Townsend's Big-Eared Bat, *Corynorhinus townsendii*. *Molecular Ecology Resources* **9:**258-260 DOI 10.1111/j.1755-0998-2008-02243.x.

**Trujillo RG, Amelon SK. 2009.** Development of Microsatellite Markers in *Myotis sodalis* and Cross-Species Amplification in *M. grisescens*, *M. leibii*, *M. lucifugus*, and *M. septentrionalis*. *Conservation Genetics* **10:**1965-1968 DOI 10.1007/s10592-009-9869-1.
