# Supplementary material for: Genetic approaches to the conservation of migratory bats: a study of the eastern red bat (Lasiurus borealis)
Source: PeerJ. 2015 May 28;3:e983. doi: 10.7717/peerj.983 (PMC4451038; doi:10.7717/peerj.983)
Supplement: Table S3 [file peerj-03-983-s003.docx]

Table S3. Diversity of microsatellite loci, including number of alleles (*N*_A_), observed (*H*_O_) and expected heterozygosity (*H*_E_), estimated null allele frequency (F_(Null)_), and allelic richness (*AR*).

| Locus | *N*_A_ | *H*_O_ | *H*_E_ | *F*_(Null)_ | *AR* |
| --- | --- | --- | --- | --- | --- |
| MS3E10 | 35 | 0.776 | 0.888 | 0.068 | 12.4 |
| MS1C01 | 41 | 0.757 | 0.836 | 0.054 | 12.3 |
| Coto_G12F_B11R | 32 | 0.923 | 0.945 | 0.012 | 17.0 |
| Cora_F11_C04 | 26 | 0.965 | 0.939 | -0.015 | 15.3 |
| IBat Ca22 | 46 | 0.82 | 0.896 | 0.044 | 14.7 |
| LboB06 | 13 | 0.687 | 0.659 | -0.020 | 4.3 |
| LboC07 | 20 | 0.703 | 0.723 | 0.018 | 6.7 |
| LboD08 | 69 | 0.835 | 0.974 | 0.076 | 23.5 |
| LboD200 | 57 | 0.93 | 0.959 | 0.015 | 20.1 |
| LboD202 | 12 | 0.717 | 0.824 | 0.068 | 7.5 |
| LboD203 | 29 | 0.863 | 0.902 | 0.020 | 11.7 |
| LboD204 | 29 | 0.817 | 0.884 | 0.040 | 11.9 |
| LboD226 | 25 | 0.704 | 0.902 | 0.123 | 12.0 |
| LboD240 | 45 | 0.908 | 0.909 | 0.000 | 15.4 |
| LboD245 | 22 | 0.817 | 0.845 | 0.015 | 8.5 |
| LboD248 | 54 | 0.944 | 0.958 | 0.007 | 19.3 |
| Mean | 34.7 | 0.823 | 0.878 |  | 13.3 |
