# Supplementary material for: Genetic approaches to the conservation of migratory bats: a study of the eastern red bat (Lasiurus borealis)
Source: PeerJ. 2015 May 28;3:e983. doi: 10.7717/peerj.983 (PMC4451038; doi:10.7717/peerj.983)
Supplement: Table S4 — Mean LnP is the mean log likelihood for a given K across runs. Other terms are defined in Earl & vonHoldt (2012). [file peerj-03-983-s004.docx]

Table S4. Structure results for various numbers of clusters (*K* = 1-10) as produced by STRUCTURE HARVESTER. Mean LnP is the mean log likelihood for a given *K* across runs. Other terms are defined in Earl & vonHoldt (2012).

| *K* | Reps | Mean LnP(*K*) | Stdev LnP(K) | Ln'(K) | \|Ln''(K)\| | Delta K |
| --- | --- | --- | --- | --- | --- | --- |
| 1 | 10 | -28351.6 | 0.9028 | - | - | - |
| 2 | 10 | -28364.6 | 3.1504 | 7 | 207.28 | 65.794887 |
| 3 | 10 | -28564.88 | 100.794 | -200.28 | 137.18 | 1.360994 |
| 4 | 10 | -28902.34 | 214.7957 | -337.46 | 1377.36 | 6.41242 |
| 5 | 10 | -30617.16 | 1198.8371 | -1714.82 | 80.04 | 0.066765 |
| 6 | 10 | -32251.94 | 2002.1587 | -1634.78 | 2655.2 | 1.326169 |
| 7 | 10 | -31231.52 | 2201.8367 | 1020.42 | 1227.64 | 0.557553 |
| 8 | 10 | -31438.74 | 333.8201 | -207.22 | 683.3 | 2.046911 |
| 9 | 10 | -32329.26 | 1685.2558 | -890.52 | 1162.02 | 0.689521 |
| 10 | 10 | -34381.8 | 5760.8588 | -2052.54 | - | - |
