# Supplementary material for: Genetic approaches to the conservation of migratory bats: a study of the eastern red bat (Lasiurus borealis)
Source: PeerJ. 2015 May 28;3:e983. doi: 10.7717/peerj.983 (PMC4451038; doi:10.7717/peerj.983)
Supplement: Table S5 [file peerj-03-983-s005.docx]

Table S5. Locus information for IMa2 analysis.

| Locus | Genome location | Sample size (chromosomes) | Relative mutation rate^A^ | Calculated^B^ mutation rate (per locus per yr) |
| --- | --- | --- | --- | --- |
| CHY | Autosomal | 164 | 0.2938 | 2.29 x 10^-6^ |
| HV2 | Mitochondrial | 160 | 1.127 | 8.77 x 10^-6^ |
| MS3E10 | Autosomal | 200 | 1.514 | 11.8 x 10^-6^ |
| MS1C01 | Autosomal | 200 | 1.009 | 7.85 x 10^-6^ |
| G12B11 | Autosomal | 200 | 2.582 | 20.1 x 10^-6^ |
| F11C04 | Autosomal | 200 | 2.399 | 18.7 x 10^-6^ |
| Ca22 | Autosomal | 200 | 1.148 | 8.93 x 10^-6^ |
| D203 | Autosomal | 200 | 1.432 | 11.1 x 10^-6^ |
| D248 | Autosomal | 200 | 7.798 | 60.7 x 10^-6^ |
| D204 | Autosomal | 200 | 1.380 | 10.7 x 10^-6^ |
| C07 | Autosomal | 200 | 0.169 | 1.31 x 10^-6^ |
| D202 | Autosomal | 200 | 0.1028 | 0.8 x 10^-6^ |
| D226 | Autosomal | 200 | 1.282 | 9.97 x 10^-6^ |
| D200 | Autosomal | 200 | 2.228 | 17.3 x 10^-6^ |
| B06 | Autosomal | 200 | 0.1406 | 1.09 x 10^-6^ |
| D08 | Autosomal | 200 | 5.395 | 42.0 x 10^-6^ |
| D245 | Autosomal | 200 | 0.6855 | 5.33 x 10^-6^ |
| D240 | Autosomal | 200 | 1.236 | 9.62 x 10^-6^ |
| Average | | | | 8.03 x 10^-6^ |

^A^Estimated from IMa2 analysis.

^B^Calculations assumed Pesole et al.’s (1999) estimate of 2.74 x 10^-8^ substitutions per site per year for the average rate of mitochondrial synonymous coding substitutions.
