# Supplementary figures and images for: Genetic approaches to the conservation of migratory bats: a study of the eastern red bat (Lasiurus borealis)
Source: PeerJ. 2015 May 28;3:e983. doi: 10.7717/peerj.983 (PMC4451038; doi:10.7717/peerj.983)

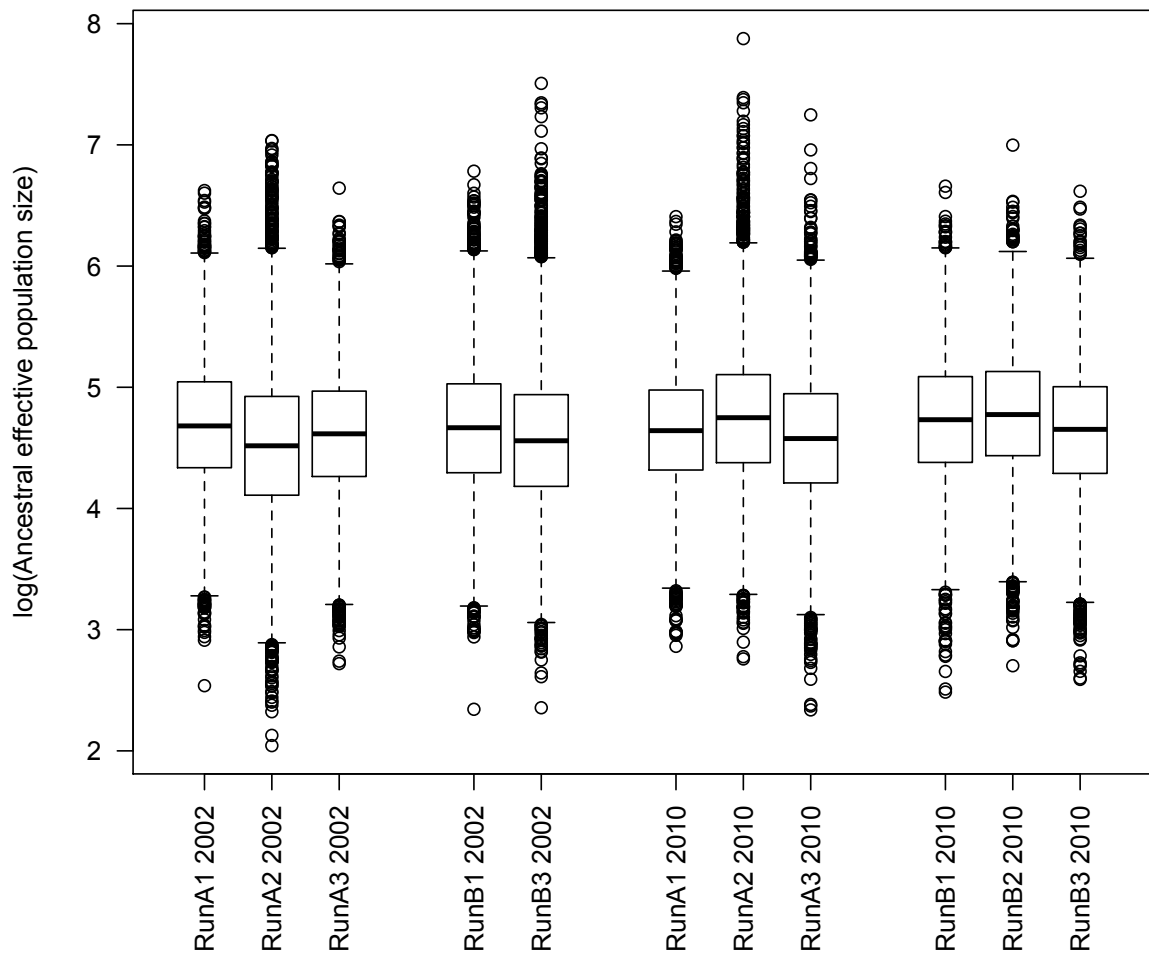

Supplement: Figure S1 — Estimates are given on the log10 scale. Datasets A and B represent different subsamples of the full dataset from each respective year. [file peerj-03-983-s006.pdf]

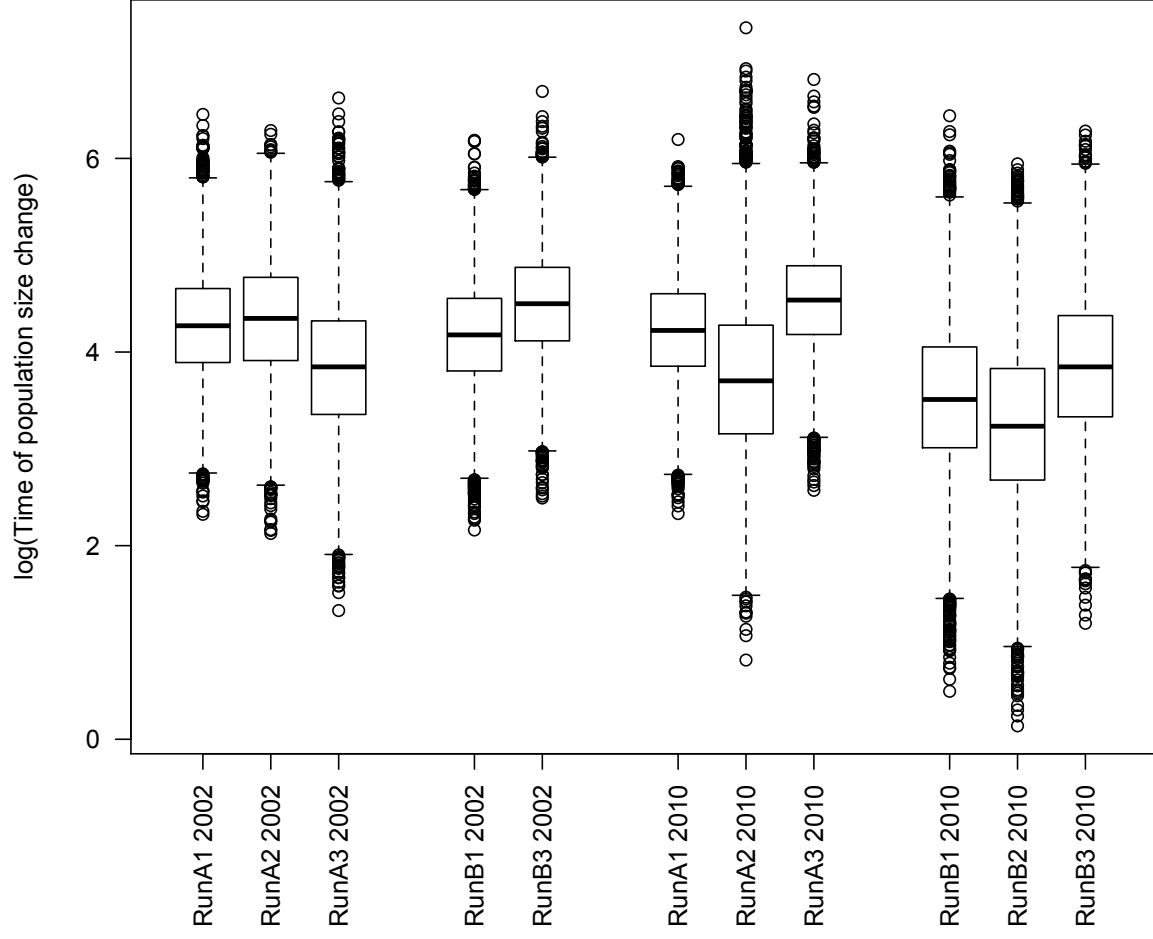

Supplement: Figure S2 — Estimates are given on the log10 scale. Datasets A and B represent different subsamples of the full dataset from each respective year. [file peerj-03-983-s007.pdf]
